# Involvement of oxytocin receptor deficiency in psychiatric disorders and behavioral abnormalities

**DOI:** 10.3389/fncel.2023.1164796

**Published:** 2023-04-20

**Authors:** Jinbao Wei, Huanrui Zheng, Guokai Li, Zichun Chen, Gengjing Fang, Jianying Yan

**Affiliations:** ^1^Department of Pharmacy, Fujian Maternity and Child Health Hospital, College of Clinical Medicine for Obstetrics and Gynecology and Pediatrics, Fujian Medical University, Fuzhou, Fujian, China; ^2^Department of Pharmacy, Ningde Municipal Hospital Affiliated to Ningde Normal University, Ningde, Fujian, China; ^3^Fujian Key Laboratory of Women and Children's Critical Diseases Research, Fujian Maternity and Child Health Hospital, Fuzhou, Fujian, China; ^4^NHC Key Laboratory of Technical Evaluation of Fertility Regulation for Non-human Primate (Fujian Maternity and Child Health Hospital), Fuzhou, Fujia, China; ^5^Department of Obstetrics, Fujian Maternity and Child Health Hospital, College of Clinical Medicine for Obstetrics and Gynecology and Pediatrics, Fujian Medical University, Fuzhou, Fujian, China

**Keywords:** oxytocin/OXTR system, oxytocin receptor deficiency, psychiatric disorders, social deficits, genetic variations

## Abstract

Oxytocin and its target receptor (oxytocin receptor, OXTR) exert important roles in the regulation of complex social behaviors and cognition. The oxytocin/OXTR system in the brain could activate and transduce several intracellular signaling pathways to affect neuronal functions or responses and then mediate physiological activities. The persistence and outcome of the oxytocin activity in the brain are closely linked to the regulation, state, and expression of OXTR. Increasing evidence has shown that genetic variations, epigenetic modification states, and the expression of OXTR have been implicated in psychiatric disorders characterized by social deficits, especially in autism. Among these variations and modifications, OXTR gene methylation and polymorphism have been found in many patients with psychiatric disorders and have been considered to be associated with those psychiatric disorders, behavioral abnormalities, and individual differences in response to social stimuli or others. Given the significance of these new findings, in this review, we focus on the progress of OXTR's functions, intrinsic mechanisms, and its correlations with psychiatric disorders or deficits in behaviors. We hope that this review can provide a deep insight into the study of OXTR-involved psychiatric disorders.

## 1. Introduction

Oxytocin (OXT), a neuropeptide produced by the paraventricular nucleus (PVN) and the supraoptic nucleus (SON) of the hypothalamus in the brain, mainly acts as a hormone in the peripheral system, which can promote milk secretion during breastfeeding, regulate uterine contraction during childbirth, relieve pain and facilitate the birth process, and promote wound healing (Carter, 2014; Kendrick et al., 2018; Sue Carter, 2018). Moreover, the OXT system in the central nervous system (CNS) plays important roles in social recognition, social behaviors, anxiety-related behaviors, and autonomic functions, such as maternal behavior, aggression, mating, attachment, and sexual behavior (Ferguson et al., 2001; Yoshida et al., 2009; Neumann and Slattery, 2016; Jones et al., 2017). In addition to the pro-social effects, OXT has been identified as a significant anti-stress and anxiolytic factor in the brain with the activation of intracellular MEK/ERK signaling *via* its target receptor (oxytocin receptor, OXTR), (Zhong et al., 2003; Aoki et al., 2014; Neumann and Slattery, 2016). Due to the promising therapeutic outcomes and applications in improving social recognition, intranasal oxytocin administration has been proposed to treat neuropsychiatric disorders characterized by deficits in social recognition, especially for autism spectrum disorder (ASD) (Anagnostou et al., 2012; Penagarikano, 2015; Rae et al., 2022).

As a G-protein-coupled receptor (GPCR), OXTR is widely expressed in multiple functional brain regions including the prefrontal cortex, amygdala, hippocampus, hypothalamus, and other regions (Kimura et al., 2003; Lin et al., 2003; Jones et al., 2017). OXTR in the central nervous system is involved in the regulation of social cognition, anxiety, depression, and other psychological behaviors or emotions, with the activation of intracellular signaling pathways, such as the mitogen-activated protein kinase (MAPK), protein kinase C (PKC), and phospholipase C (PLC) signaling (Fitts et al., 2003; Zingg and Laporte, 2003; Yoshida et al., 2009; Carter, 2014; Kendrick et al., 2018). Recent studies have found that rodents bearing deficits of either OXTR or OXT by gene knockout displayed autism-related behaviors and significant impairments in social cognition, besides increased aggressive behavior (Winslow and Insel, 2002; Takayanagi et al., 2005; Pobbe et al., 2012; Sala et al., 2013), indicating that OXTR functions as a crucially functional gene in the regulation of social cognition and behaviors. Furthermore, epigenetic down-modulation of OXTR was related to high levels of separation anxiety and arousal in response to social separation in rhesus macaques experiencing maternal deprivation stress during the neonatal period (Baker et al., 2017). In humans, individuals with neurodevelopmental disorders including obsessive-compulsive disorders (OCDs) and ASD exhibited abnormal DNA methylation modification in the OXTR gene (Wu et al., 2005; Siu et al., 2021; Bey et al., 2022). Reduced OXTR gene expression in the temporal cortex and binding sites in the vermis were found in schizophrenia patients compared to healthy controls (Uhrig et al., 2016). Moreover, patients with ASD, post-traumatic stress disorder (PTSD), attention-deficit hyperactivity disorder (ADHD), or other psychiatric disorders showed conspicuous variations in the OXTR gene (Gregory et al., 2009; Kalyoncu et al., 2019; Cao et al., 2020; Kimura et al., 2020). These studies suggested that deficits or variations of OXTR may be involved in many psychiatric disorders.

Consequently, individual differences in the gene expression, variability, or epigenetic modification of OXTR that lead to a dysfunction of OXT signaling may imply or contribute to different behavioral expressions and phenotypes of mental health in humans (Uhrig et al., 2016; Bos, 2017; Cataldo et al., 2018; Kimura et al., 2020; Ji et al., 2021). This review aimed to discuss the roles of OXTR in social behavior and cognition, the signaling pathway network of OXTR regulation, the involvement of genetic variation, epigenetic modification, and expression of OXTR in psychiatric disorders, as well as the therapeutic applications of the OXT value in the treatment of psychiatric disorders.

## 2. The gene structure and expression of OXTR in humans and mice

**Figure 1:**
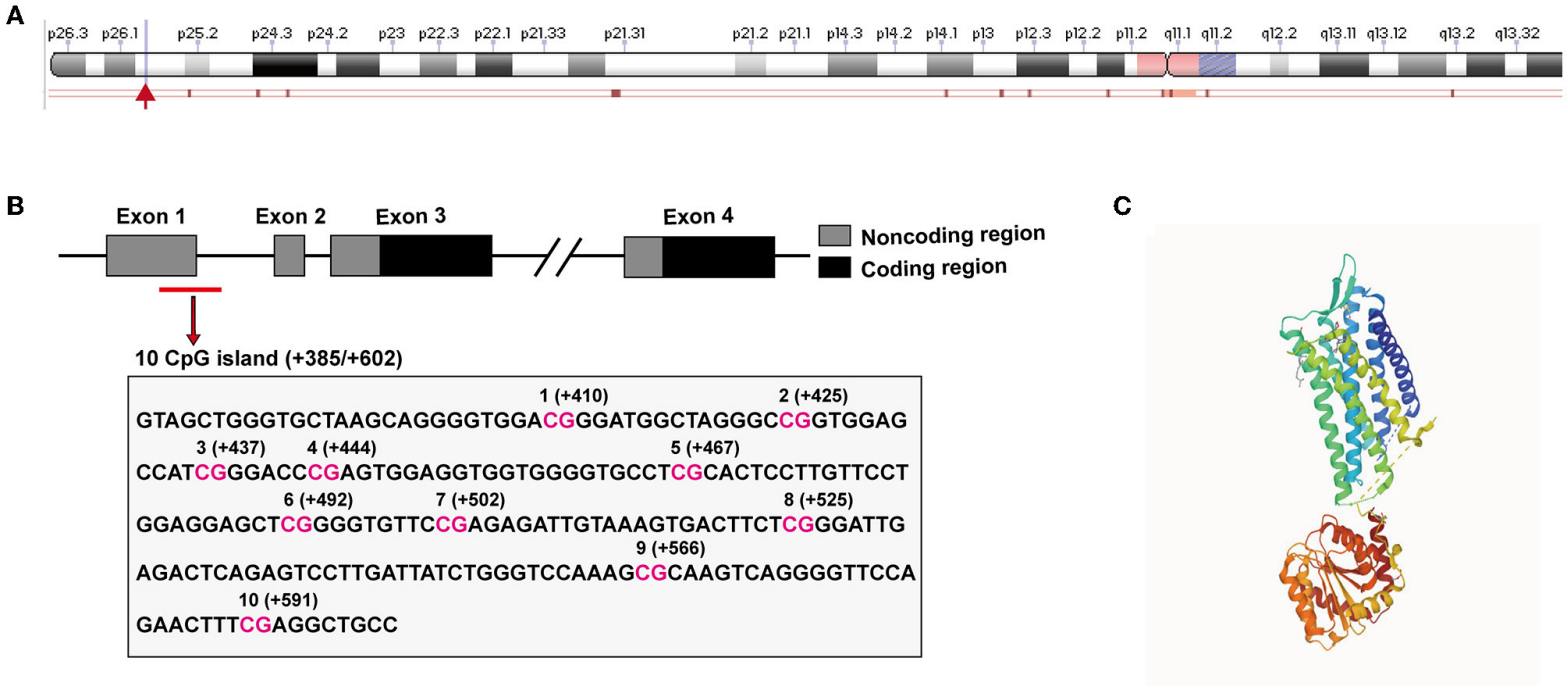
OXTR gene structure and tissue expression in humans. **(A)** The location of the OXTR gene on the human chromosome 3 (The information was obtained from the NCBI: OXTR gene 5021) is labeled with a red arrow. **(B)** Gene schematic of OXTR in humans. Exons are indicated by boxes (coding, black; untranslated, gray). Introns are shown with solid lines. The black box below the OXTR gene denotes CpG islands which function as DNA hypermethylation sites. The figure was generated using information obtained from Cho et al. (2019). **(C)** X-ray crystal structure of OXTR protein (PDB database: 6TPK).

The oxytocin receptor is encoded by a single gene (human OXTR gene, gene ID: 5021), which is localized to chromosome 3 p25–p26 in humans and structurally spans ~17 kilobases (kb), and contains four exons and three introns (Inoue et al., 1994; Simmons et al., 1995; Wu et al., 2005) (Figure 1A). Within the gene sequence, the OXTR gene contains many CpG islands that are thought to be important regions of the epigenetic regulation of OXTR DNA hypermethylation (Gregory et al., 2009). Moreover, OXTR DNA hypermethylation is closely linked to the downregulation of OXTR in humans (Kusui et al., 2001). As shown in Figure 1B, there are 10 CpG sites within the 217-bp fragment of the human OXTR gene (starting from +385 to +602 bp at the transcription start site) (Cho et al., 2019). It was found that adults who suffered from low maternal care in childhood bore greater DNA methylation in peripheral whole blood compared with those who experienced high maternal care in childhood, suggesting that early life events were associated with OXTR DNA methylation (Unternaehrer et al., 2015; Danoff et al., 2021; Ramo-Fernandez et al., 2021).

**Figure 2:**
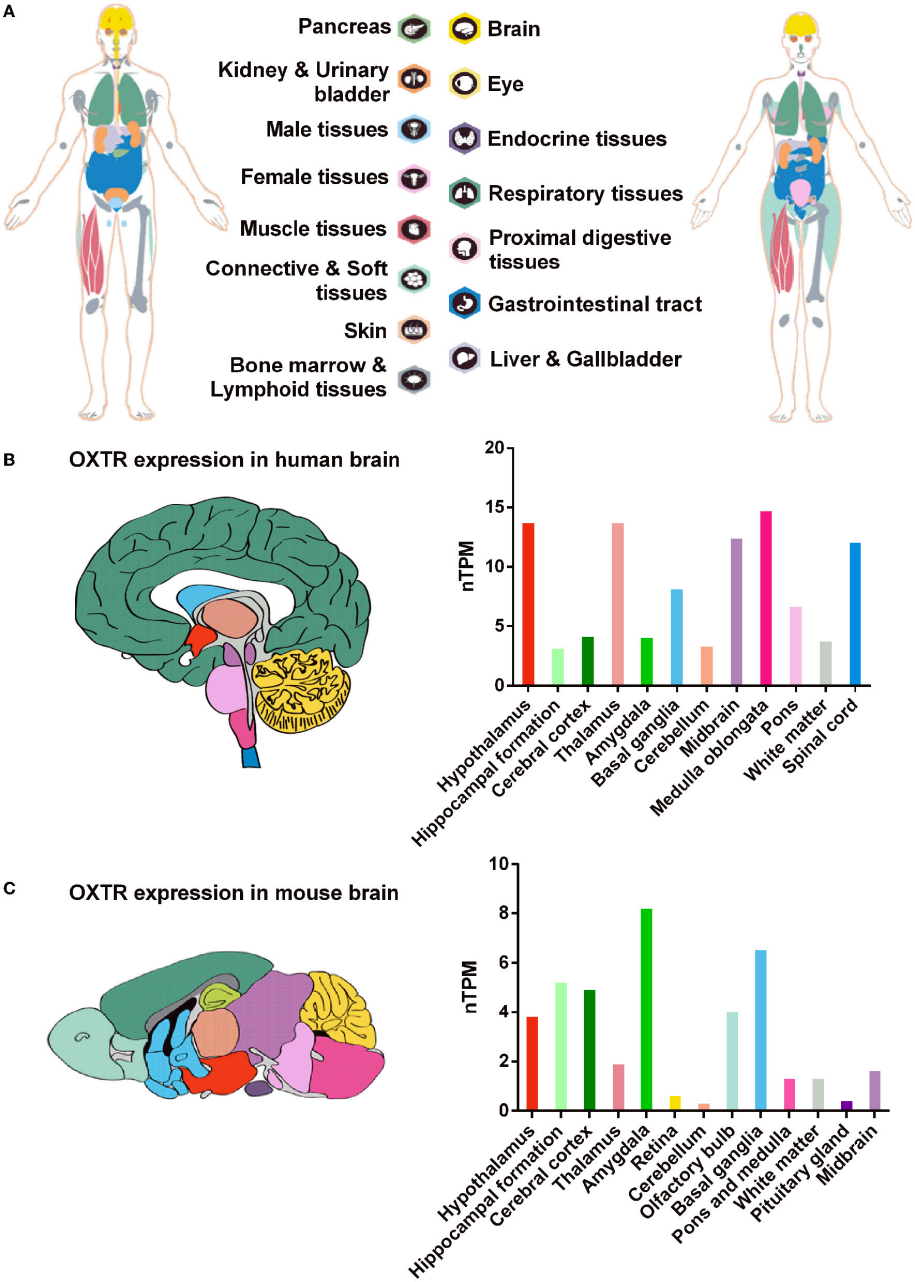
Tissue distribution of OXTR expression in humans and mice. **(A)** The OXTR expression in human tissues or organs. **(B, C)** The relative quantitative RNA expression of OXTR was shown by nTPM (normalized expression) in human and mouse brains. Data were obtained from The Human Protein Atlas, HPA Human brain dataset, and HPA Mouse brain dataset: https://www.proteinatlas.org/.

Oxytocin receptor is a 388-amino-acid polypeptide and is a very highly conservative rhodopsin-type (class I) G-protein-coupled receptor (GPCR) with typical seven transmembrane domains across species (Figure 1C) (Kimura et al., 1992). In humans, OXTR is extensively expressed in a great variety of tissues, ranging from peripheral tissues, such as the ovary, adrenal, endometrium, prostate, and lung to the brain (Figure 2A). OXTR was found to be highly expressed in the important brain regions of humans and mice, including the PFC, hippocampus, hypothalamus, amygdala, and others (Figures 2B, C) (Busnelli and Chini, 2018). As a typical GPCR, OXTR in the brain is expressed on the cytomembrane of neuronal cells and astrocytes (Tan et al., 2019) and transmits extracellular signaling *via* the activation of intracellular phospholipase C (PLC), the mitogen-activated protein kinase (MAPK), protein kinase D1 (PKD1), or other pathways (Fitts et al., 2003; Zingg and Laporte, 2003; Carter, 2014; Wang et al., 2022). By coupling the ligand OXT to OXTR, this OXT/OXTR system plays critical roles in the regulation of social recognition and other behaviors or emotions in the brain. Widely expressed OXTR in the brain is significant to determine the various responses in different brain regions and neuronal cells.

## 3. The regulatory effects of the OXT/OXTR system in the CNS of humans and animals

**Figure 3:**
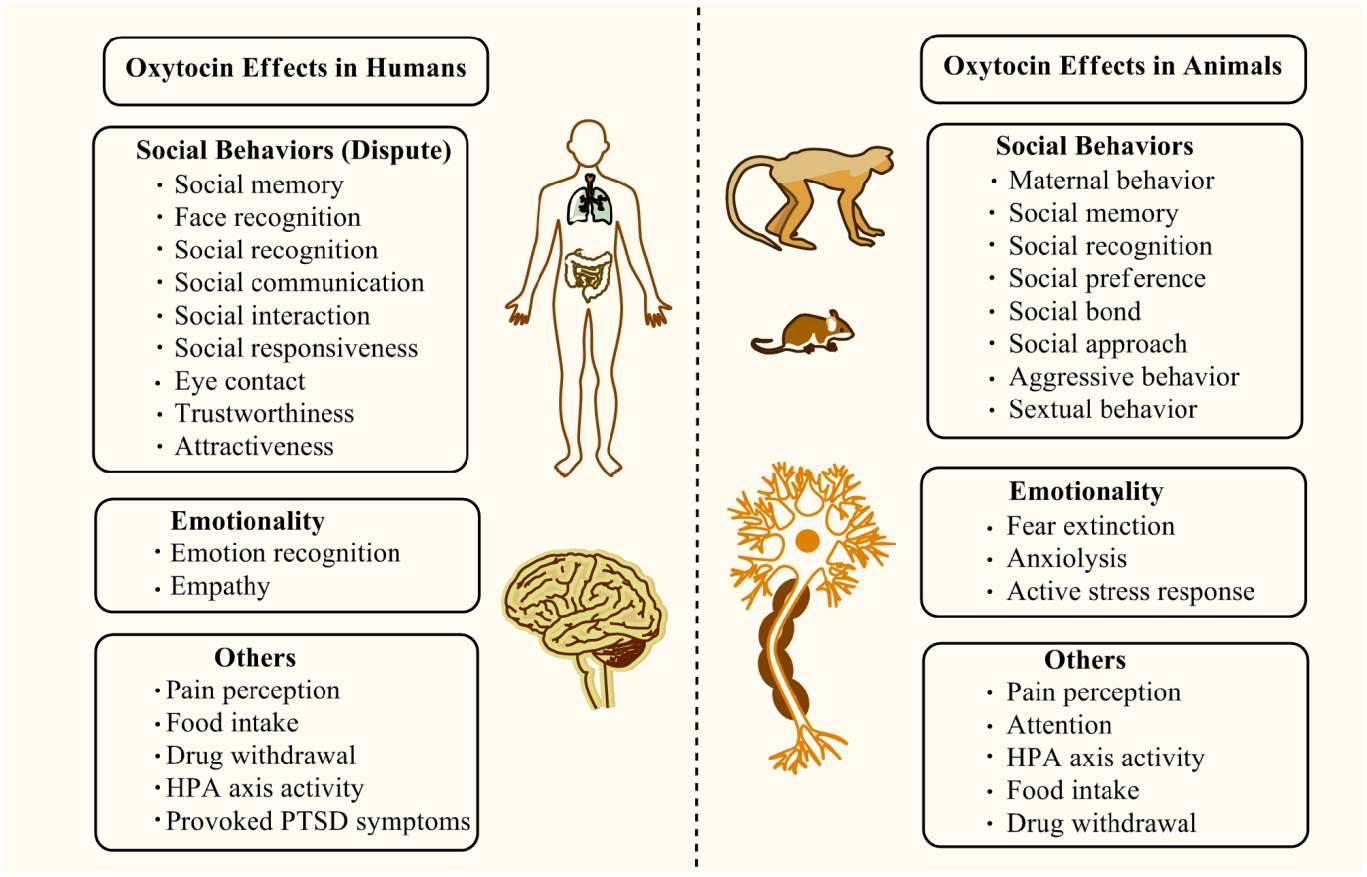
The main regulatory effects of the OXT/OXTR system on social behaviors, emotionality, and other functions are found in humans and animals.

In addition to the support of growth during development or uterine contraction in the peripheral system, OXT acting by its single receptor subtype, the OXTR serves as a unique hypothalamic neuropeptide/neurotransmitter with a powerfully broad profile of behavioral and neurological effects in the CNS and mediates a diverse range of behaviors and CNS functions (Figure 3) (Jurek and Neumann, 2018). Among them, the most studied effects of OXT in the CNS are involved in social behaviors. In humans, OXT was found to improve social deficits, such as face recognition and social responsiveness, in ASD or other neurodevelopmental disorders (Anagnostou et al., 2012; Guastella et al., 2015b; Penagarikano, 2015; Yatawara et al., 2016). Parker et al. found that the intranasal oxytocin treatment enhanced social abilities in children with ASD, especially in the ASD individuals with the lowest pretreatment OXT concentrations (Parker et al., 2017). A posttreatment increase in the blood OXT concentrations was detected, which might underlie the improvement of OXT in ASD. However, several randomized controlled trials showed that there was no benefit for the youth with ASD who received intranasal OXT in comparison to the placebo controls, indicating that OXT could not ameliorate the social behavioral deficits in ASD (Dadds et al., 2014; Guastella et al., 2015a; Yamasue et al., 2020). Additionally, a recent study published in the N Engl J Med journal found that intranasal OXT in children and adolescents with ASD showed no significant difference in the improvements of social or cognitive functions compared with the placebo group (Sikich et al., 2021). Thus, these studies have given rise to a contrary result about the OXT therapeutic outcomes in social behaviors.

Despite the dispute about its clinical effectiveness in ASD, an OXT administration in mouse models of ASD, Cntnap2^−/−^, Chd8^+/Δ*S*^, and POGZ^WT/Q1038R^ could reverse the social behavioral deficits (Cherepanov et al., 2021; Kitagawa et al., 2021; Choe et al., 2022). Other studies showed that OXT restored abnormal neuronal morphology and synaptic plasticity deficits in the medial prefrontal cortex and hippocampus; rescued attention, social recognition, and social memory deficits in Shank3-deficient rats, which were commonly used as animal models of ASD (Harony-Nicolas et al., 2017; Reichova et al., 2020); and improved or regulated social preference, social bond, social approach, and sexual behavior, as well as decreased stress response, anxiety, and aggression behavior in animals (Braida et al., 2012; Chang and Platt, 2014; Wei et al., 2020). In addition to the pro-social and anti-stress effects, OXT reduced anxiety behaviors in animals, acting as an anxiolytic nonapeptide *in vivo* (Ring et al., 2006). In humans, intranasal oxytocin treatment in PTSD patients decreases provoked total symptoms in a randomized controlled trial, especially in avoidance, showing positive effects on the intensity of provoked PTSD symptoms (Sack et al., 2017). In addition, other studies in humans indicated that OXT could increase social memory, social recognition, gaze-to-eye regions, perceptions of trustworthiness and attractiveness, empathy, and cooperation within one's own group (Kosfeld et al., 2005; Baumgartner et al., 2008; Guastella et al., 2008a,b; Unkelbach et al., 2008; Di Simplicio et al., 2009; Ditzen et al., 2009; Keri and Benedek, 2009; Theodoridou et al., 2009; De Dreu et al., 2010; Domes et al., 2010; Fischer-Shofty et al., 2013; Bartz et al., 2019) and improve emotion recognition, social communication, and interaction in youth with ASD (Guastella et al., 2010). Hence, due to the inconsistency between humans and animals findings, further adequate studies are necessary to elucidate whether OXT has therapeutic potential for social deficits or others involved in neurodevelopmental disorders.

## 4. The intracellular signaling of OXT/OXTR

**Figure 4:**
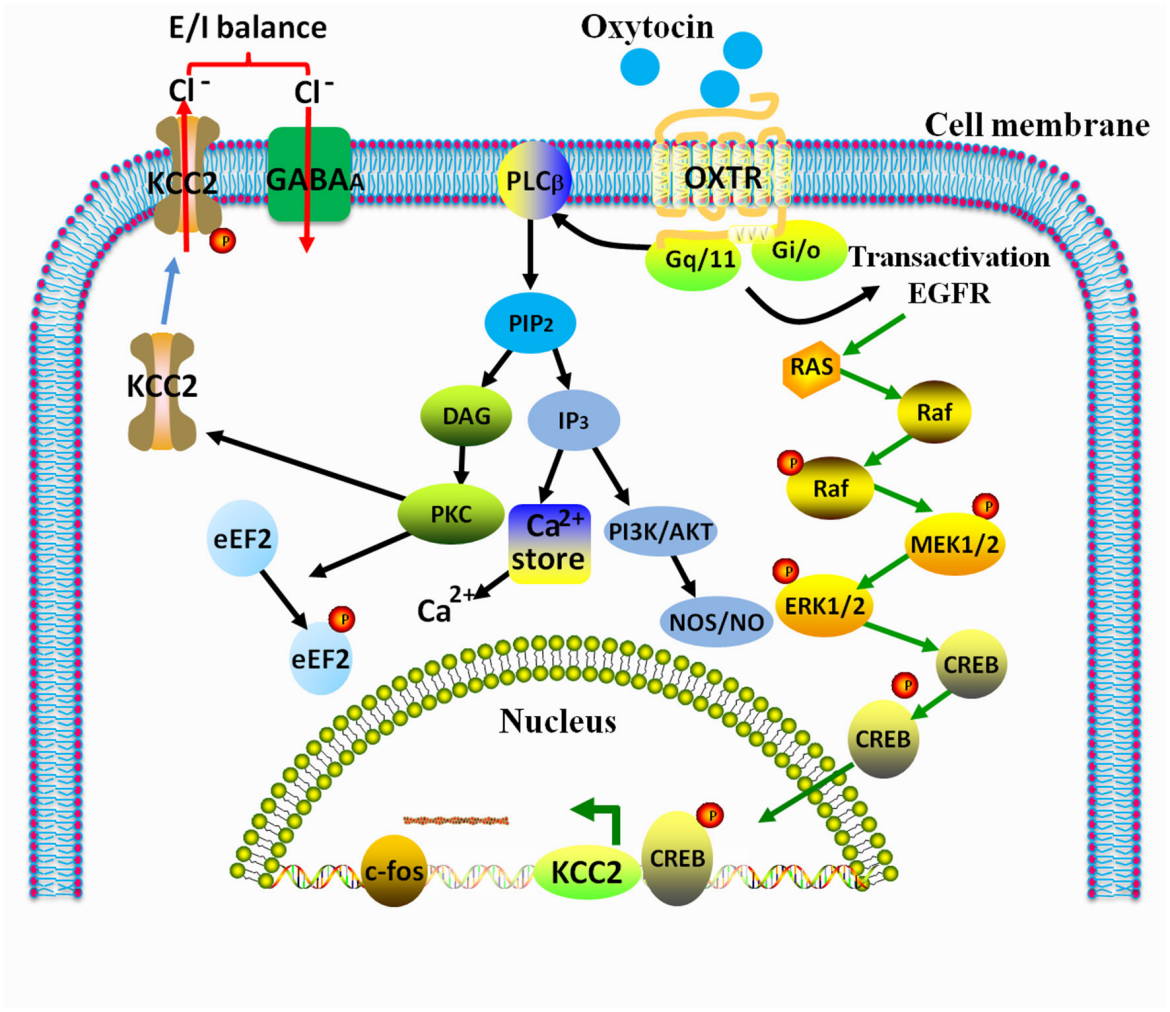
Relevant signaling pathways of OXT/OXTR in the central nervous system regulate gene transcription and expression and induce LTP, E/I balance, and behavioral responses (Busnelli and Chini, 2018). *E/I* excitation/inhibition, *PKC* protein kinase C, *LTP* long-term potentiation, *eEF2* eukaryotic elongation factor 2, *KCC2* K^+^-Cl^−^ co-transporter 2, *PLC*_β_ phospholipase C_β_, *ERK1/2* extracellular signal-regulated kinase1/2, *MEK1/2* mitogen-activated protein kinase kinase1/2, *CREB* cAMP response element binding protein, *PI3K* phosphoinositide (PI)3-kinase, *EGFR* epidermal growth factor receptor, *DAG* diacylglycerol, and *IP*_3_ inositol triphosphate.

As a GPCR membrane protein, OXTR can sense and receive extracellular signals and then regulate intracellular effects. OXT bonding to OXTR facilitates the transduction of OXTR-coupled signaling pathways into the cells and even the nucleus, thereby mediating a variety of physiological effects and behaviors in response to stressors. Many recent studies have shown that the intracellular effectors or downstream signaling of OXT/OXTR mainly include extracellular regulated kinase/microtubule-associated protein kinase (ERK/MAPK) cascade, eEF2 phosphorylation, NO production, PLCβ/PKC cascade, KCC2 phosphorylation and expression, and the excitatory-to-inhibitory GABA switch (Figure 4).

### 4.1. ERK/MAPK

The mitogen-activated protein kinase pathway is one of the most important intracellular signaling pathways activated by OXT/OXTR (Busnelli and Chini, 2018). MAPK-related proteins are highly conserved and ubiquitous in eukaryotic cells. There are four well-characterized MAPK sub-families in mammalian cells: p38 MAPK; c-Jun N-terminal kinase (JNK, also known as stress-activated protein kinase-1, SAPK1); ERK1/2, also known as p42/44 MAPK; and ERK5 (also known as big mitogen-activated protein kinase) (Sun and Nan, 2016). These signaling pathways can be activated by kinases recruited by various extracellular and intracellular stimuli including hormones, cytokines, peptide growth factors, neurotransmitters, and cellular stressors such as endoplasmic reticulum stress and oxidative stress (Kim and Choi, 2010). Serving as a critical transduction signaling pathway, the ERK/MAPK cascade plays important roles in gene expression, cellular proliferation and differentiation, migration, senescence, apoptosis, and neurodevelopment in the CNS, which could influence the cellular responses and behavioral consequences (Sun et al., 2015; Iroegbu et al., 2021). Due to the pivotal functions of the ERK/MAPK cascade in neurodevelopment, its dysfunction has been involved in various psychiatric disorders, especially in neurodevelopmental disorders (Iroegbu et al., 2021).

It has been demonstrated that conditional knockout of ERK2 in the CNS resulted in increased aggressive behavior, decreased nesting ability, inadequate maternal care, and lower levels of social interaction and sociability in mice, suggesting that the ERK/MAPK pathway was implied in regulating social behavior (Satoh et al., 2011). Furthermore, activation of OXTR could lead to the phosphorylation of ERK and cAMP-responsive element binding (CREB) protein, induce long-term potentiation (LTP), and then lead to durable improvements in spatial memory function in the mouse hippocampus (Tomizawa et al., 2003). Studies have shown that OXT in the hypothalamus exerted anxiolytic effects *via* the activation of OXTR/ERK/MAPK, activated the phosphorylation of the nuclear transcription factor CREB, and then regulated the gene transcription of corticotropin hormone-releasing factor (Crf) together with CREB-regulated transcription coactivator 3 (CRTC3). ERK1/2 blocker could inhibit OXT-induced neuronal action potentials (Jurek et al., 2015), which indicated that ERK/MAPK was a key downstream of OXT/OXTR in regulating intracellular signaling.

### 4.2. Gq/PLC/PI3K/NO signaling

Nitric oxide (NO) is an important signaling molecule that acts as an endogenous gasotransmitter in the human body. Due to the existence of neuronal nitric oxide synthase (nNOS), NO is widely pervasive in the nervous system, regulating activity-dependent control of intrinsic neuronal excitability, synaptic plasticity (long-term depression, LTD; long-term potentiation, LTP), and other various biological functions and processes in the brain (Steinert et al., 2010). Previous research showed that OXT induced the release of NO in the PVN of the hypothalamus in rats and that it led to yawning and penile erection behaviors (Melis et al., 1997). Moreover, Melis MR et al. also found that a specific OXTR antagonist could prevent the OXT-induced yawning and penile erection behaviors and inhibited the OXT-induced increase of ${\text{NO}}_{2}^{-}$ (Melis et al., 1997). A recent study by Gong et al. showed that the peripheral anti-nociceptive effects of OXT were mediated by Ca^2+^/nNOS/NO/KATP signaling (Gong et al., 2015). In vascular endothelial cells, it was found that OXTR activation led to the calcium mobilization and phosphorylation of endothelial nitric oxide synthase (eNOS) by Gq/PLC/PI3K/AKT signaling (Cattaneo et al., 2008; Busnelli and Chini, 2018). These studies provided a clue that NO served as a downstream effector molecule of OXT.

### 4.3. eEF2 phosphorylation

In OXT-induced changes in protein phosphorylation, eukaryotic elongation factor 2 (eEF2), which functions as an important regulator of ubiquitous protein synthesis, is a new downstream target of OXTR in the human body (Devost et al., 2008). It has been suggested that OXT-induced intracellular effectors can be regulated by the Gq/PKC-mediated phosphorylation of eEF2 and that OXTR was involved in the regulation of the neuronal balance of excitatory/inhibitory (E/I) through eEF2 signaling (Devost et al., 2008; Heise et al., 2017). OXTR deficiency or a decrease in the eEF2 activity could significantly reduce GABAergic synaptic transmission in neurons (Sala et al., 2011; Leonzino et al., 2016; Heise et al., 2017). Thus, the effect of OXTR on GABAergic synaptic transmission might be partly mediated by the eEF2 phosphorylation signaling (Busnelli and Chini, 2018).

### 4.4. KCC2

Postnatal brain development requires a coordinated balance of excitation and inhibition to shape the correct neuroregulatory network (Leonzino et al., 2016; Busnelli and Chini, 2018). GABA produces membrane excitatory depolarization through the activation of GABA_A_ receptors (GABA_A_Rs). The intracellular chloride concentration is regulated by the expression of the two Cl^−^ transporters, namely NKCC1 (a Cl^−^ importer) and KCC2 (a Cl^−^ exporter). During the early period of development, intracellular chloride concentration (${\text{Cl}}_{\text{i}}^{-}$) in the neurons is higher than the extracellular concentration (${\text{Cl}}_{\text{O}}^{-}$). With the subsequent expression of KCC2, the intracellular chloride concentration decreases, and then the excitatory-to-inhibitory GABA transition (GABA switch) is completed. This GABA switch occurs within 1 week in rodents (Valeeva et al., 2013). The normal development of the brain depends on the timing of the GABA switch from depolarization to hyperpolarization (Tang et al., 2021).

Abnormal KCC2 function was associated with a variety of psychiatric disorders, including social impairment and schizophrenia (Kahle et al., 2015). Marianna Leonzino et al. found that the knockout of the OXTR gene could induce the inhibition of KCC2 expression and phosphorylation, and OXTR-null delayed excitatory-to-inhibitory GABA switch and altered E/I balance in hippocampal neurons from OXTR-null mice, and OXT could increase KCC2 phosphorylation and KCC2 insertion in the membrane through Gq/PKC signaling (Leonzino et al., 2016). Furthermore, a study from Ben Ari group suggested that OXT or a selective NKCC1 inhibitor (bumetanide) at birth rescued the behavioral dysfunctions in two animal models of autism that showed deficits in the GABA switch (Eftekhari et al., 2014; Tyzio et al., 2014). These findings indicated that OXT/OXTR might regulate the GABA switch and E/I balance *via* the Gq/PKC/KCC2 pathway.

## 5. Behavioral deficits during development due to dysregulated OXTR

The OXT/OXTR signaling is implicated in a variety of normal behaviors, including social interaction, anxiety-related behavior, depression, maternal behavior, aggression, mating, attachment, and sexual behavior (Ferguson et al., 2001; Yoshida et al., 2009; Neumann and Slattery, 2016; Jones et al., 2017). As a result, dysfunctions or perturbations of OXT/OXTR signaling have been shown to contribute to behavioral deficits during development. In Table 1, we summarized the related behavioral deficits due to aberrant OXT/OXTR signals.

**Table 1:** Summary of behavioral implications of OXT/OXTR deficiency.

**Functions**	**Model**	**Effects**	**OXTR deficiencies**	**References**
Social interaction (sociability and social novelty)	Mice (male and female)	Pervasive social deficits	Null mutation in the OXTR gene (OXTR^−/−^)	Takayanagi et al., 2005
	Mice (male)	Impaired sociability and preference for social novelty	Heterozygous mutation in the OXTR gene (OXTR^+/−^)	Sala et al., 2011, 2013
Aggressive behavior	Mice (male)	Increased aggressive behavior	Null mutation in the OXTR (OXTR^−/−^) or OXT (OXT^−/−^) gene	Takayanagi et al., 2005; Sala et al., 2013
	Mice (male and female)	Increased aggression in male mice, but not in females	Specific OXTR deletion in raphe serotonin neurons	Pagani et al., 2015
Long-term social recognition memory	Mice (male)	Impaired long-term social recognition memory but normal sociability and preference for social novelty	Conditional deletion of forebrain or hippocampal CA2/CA3a - excitatory neurons	Lin et al., 2018
Maternal behavior	Mice (females)	Impairments in the initiation of maternal behavior	Total body OXTR gene knockout (OXTR^−/−^)	Rich et al., 2014

### 5.1. Social interaction

The oxytocin receptor is an essential GPCR that regulates various behaviors in mammals, especially the social behavior, dysregulation, or deficiency of OXTR, which is involved in impaired social interaction in animal models (Liu et al., 2021). To investigate the functions of OXTR in mammals, researchers have established multiple OXTR gene-edited models. It was reported that the mice carrying a null mutation in the OXTR gene (OXTR^−/−^) displayed normal parturition and fertility or reproductive behavior but exhibited pervasive social deficits and elevated aggressive behavior, as well as in lactation and maternal nurturing, showing that OXTR plays a developmental role in shaping social abilities and aggression (Takayanagi et al., 2005). A similar study found that the OXTR gene knockout could induce autistic-like behaviors, including deficits in sociability and cognitive flexibility and impairment of preference for social novelty in OXTR^−/−^ mice, which were also found in heterozygous mice (OXTR^+/−^) having an approximate 50% decrease of OXTR expression in all of the detected brain regions (Sala et al., 2013). Moreover, the impaired behaviors in OXTR^+/−^ mice, including the preference for social novelty and sociability, could be rescued by the intracerebral administration of OXT or its analog Thr^4^Gly^7^OT (TGOT), and the effects could be blocked by OXTR antagonist (Sala et al., 2011, 2013). Blockade of OXTR by local infusion (icv) of an OXTR antagonist in the lateral septum and the medial amygdala significantly impaired the maintenance of social memory and social discrimination abilities in both rats and mice during the social tests, demonstrating that OXTR is implicated in mediating social behavior (Lukas et al., 2013). These findings suggest that OXTR acts as a critical gene in mediating social interaction and that partial inactivation or reduction of expression in OXTR could induce deficits in social behaviors that may be involved in neurodevelopmental psychiatric disorders characterized by the impairment of various social behaviors.

### 5.2. Aggressive behavior

Aggressive behavior is a flexible and hostile response for mammals, especially for males, during social interactions (Kelly and Wilson, 2020). Increased aggressive behavior or violence is implicated in some psychiatric disorders, such as schizophrenia and bipolar disorder (Volavka, 2013). Although the mechanisms underlying the aggression involved in psychiatric disorders remain unclear, emerging evidence showed the involvement of the OXT/OXTR signal in aggression (Takayanagi et al., 2005; Sala et al., 2011, 2013). Knockout of OXTR could induce significantly elevated aggressive behavior and deficits in social behavior in OXTR^−/−^ mice (Takayanagi et al., 2005; Sala et al., 2013). Unlike the null genotype, the OXTR^+/−^ mice displayed normal cognitive flexibility and aggression, which indicated that the haploinsufficiency of OXTR was sufficient to impair social behavior and that whereas, the deficits in cognitive flexibility and aggression required the entire deficiency of the OXTR gene (Sala et al., 2011, 2013). Interestingly, a high level of aggression was also found in OXT^−/−^ mice who were offspring of OXT^−/−^ dams, but not those of OXT^+/−^ dams (Takayanagi et al., 2005). Moreover, specific OXTR deletion in raphe serotonin neurons could induce aggressive behavior in male mice only, but not in female mice (Pagani et al., 2015). These results prove that the OXT/OXTR system acts as a key regulator of aggressive behavior.

### 5.3. Long-term social recognition memory

Social recognition memory is a basic survival ability for mammals to correctly recognize conspecifics, mates, and potential enemies, the impairment of which is associated with social neurobehavioral and developmental disorders (Chiang et al., 2018; Wang et al., 2022). The hippocampus in humans is considered to be a crucial brain region that is responsible for social recognition and memory. Evidence showed that the conditional deletion of hippocampal CA2/CA3a or forebrain-excitatory neurons led to the impairment of long-term social recognition memory in adult male mice without affecting the sociability, preference for social novelty, and anxiety-like behavior assessed by the novelty-suppressed feeding, elevated plus maze, and open-field tests (Lin et al., 2018). Additionally, OXTR deletion in the CA2/CA3a neurons resulted in a decreased complexity of basal dendritic arbors of CA2 pyramidal neurons and a deficit in the induction of long-term potentiation at the synapses between the CA2 and entorhinal cortex pyramidal neurons, without affecting long-term depression (Lin et al., 2018). Those findings may underlie the defect of long-term social recognition memory induced by the OXTR deficiency in the hippocampal CA2/CA3a excitatory neurons of mice. Thus, OXTR in the CA2/CA3a may serve as a target for the treatment of social recognition deficits identified in patients with neuropsychiatric disorders.

### 5.4. Maternal behavior

It has been reported that sufficient maternal care in early life is particularly crucial for brain development, social recognition, and subsequent behaviors in the adult (Cater and Majdic, 2022). Maternal behavior in mammals is an instinct that is regulated by endocrine and neural systems, especially by the OXT/OXTR system. In addition to its roles in parturition and lactation, OXT acting through its receptor subtype, OXTR, serves as a hormone of love during social interaction and promotes and strengthens the relationship between the mother and baby. Hence, it is predictable that the deficiency of OXTR may be involved in the perturbation of maternal behavior. To prove this hypothesis, Rich et al. generated a total knockout of OXTR mice (OXTR^−/−^) and found that OXTR^−/−^ dams showed significantly increased pup abandonment behavior in comparison to the controls (Rich et al., 2014; Lin et al., 2018).

### 5.5. Anxiety and depression

The OXT/OXTR system mediated anxiety-like behavior and response to stress in mammals (Neumann and Slattery, 2016). OXT also functioned as an anxiolytic and anti-stress factor in the brain, and OXT treatment in adolescent rats could promote social behaviors in adulthood and contribute to increasing OXT levels in plasma (Suraev et al., 2014). It is easy to infer that the dysfunction of OXTR may be implicated in anxiety-like or depression-like behaviors. However, it was proved that total OXTR knockout (OXTR^−/−^) or local knockout in the forebrain (OXTR^FB/FB^) did not affect the anxiety-like behavior and sucrose intake that was used to test the depression-like behavior. Additionally, the OXTR^−/−^ dams in the postpartum period had no predominant anxiety- and depression-like behaviors (Rich et al., 2014; Lin et al., 2018). Specific local OXTR knockout in the raphe serotonin neurons was not sufficient to induce anxiety-like behavior in male mice (Pagani et al., 2015), which was consistent with others' studies (Rich et al., 2014; Lin et al., 2018). These findings show that the deficiency of OXTR is inadequate to induce anxiety-like and depression-like behaviors.

## 6. The involvement of genomic, epigenetic modification, and gene expression of OXTR in psychiatric disorders

Increasing evidence warranted that aberrant OXT/OXTR signaling is an essential factor in the neuropathology of some neurodevelopmental disorders (Siu et al., 2021). Dysregulation of the OXTR gene in a variety of psychiatric disorders has enabled researchers to investigate the epigenetic state of OXTR in psychiatric patients (Gregory et al., 2009; Almeida et al., 2022). Variations in the OXTR gene (e.g., OXTR gene polymorphisms) have been proven to be associated with a variety of psychiatric disorders or alterations in brain function in humans. However, the mechanisms underlying the dysregulation of OXT/OXTR signaling in neurodevelopmental disorders remain unclear. Nonetheless, here, we summarized the aberrant OXT/OXTR signaling in the pathogenesis of the following psychiatric disorders or alterations in brain function (see Table 2).

**Table 2:** Summary of aberrant OXTR in psychiatric disorders and alterations of brain volumes.

**Psychiatric disorders**	**Samples**	**Genetic features and Behavioral phenotypes**	**Variations of OXTR**	**References**
Autism Spectrum Disorder	Human saliva, peripheral blood, and temporal cortex tissue	ASD individuals displaying high DNA methylation values in OXTR exhibited more social problems and lower IQ, correlated with symptom severity and brain functional connectivity	DNA methylation in OXTR gene	(Gregory et al., 2009; Andari et al., 2020; Danoff et al., 2021; Siu et al., 2021)
	Human temporal cortex tissue	ASD individuals showed a decrease in OXTR expression	Decreased OXTR mRNA expression	(Gregory et al., 2009)
	Human peripheral blood	ASD individuals exhibited many SNPs in OXTR gene	OXTR SNPs including rs2268491, rs7632287, rs237897, rs13316193, rs237889, rs2268494, rs237887, rs53576 and rs2254298	(Wu et al., 2005; Lerer et al., 2008; LoParo and Waldman, 2015)
Borderline Personality Disorder	Human peripheral blood	BPD patients had higher frequencies in rs53576 AA and rs237987 AA genotype	OXTR genetic polymorphisms in rs53576 and rs237987	(Hammen et al., 2015; Zhang et al., 2020)
Attention Deficit/Hyperactivity Disorder	Human	ADHD children with CT/TT genotype performed lower on the facial emotion recognition task	OXTR genetic polymorphism in rs4686302	(Kalyoncu et al., 2019)
	Human	ADHD individuals exhibiting high DNAm values in OXTR showed more social problems and lower IQ	DNA methylation of OXTR	(Siu et al., 2021)
	Human	rs53576 AA genotype was correlated with better social cognitive ability	OXTR genetic polymorphism in rs53576	(Park et al., 2010)
Schizophrenia	Human temporal cortex, vermis	Schizophrenia patients showed downregulation of OXTR mRNA in the temporal cortex and a decrease in receptor binding in the vermis	OXTR gene expression and binding sites in different brain regions	(Uhrig et al., 2016)
	Human peripheral blood	Significant associations of OXTR SNPs rs53576 with general psychopathology and rs237902 with negative symptom scores in schizophrenic patients; significant associations of OXTR SNPs rs53576(A > G) and rs237885(T > G) with a diagnosis of schizophrenia	OXTR genetic polymorphisms in rs237885, rs53576 and rs237902	(Montag et al., 2013)
Post-traumatic stress disorder	Human peripheral blood	G allele in OXTR rs53576 was related with PTSD symptoms	OXTR genetic polymorphism in rs53576	(Cao et al., 2020)
	Human peripheral blood	Higher DNA methylation was found at the two CpG-sites of OXTR (CpGs Chr3:8809437, Chr3:8809413) in female PTSD	DNA methylation in OXTR gene	(Nawijn et al., 2019)
Alterations of brain volumes	Human umbilical cord blood, saliva, peripheral blood	Smaller hippocampal volumes were associated with rs53576 AA and rs2254298 AA; rs2254298 AA was relevant to reduced bilateral amygdalar volumes	OXTR genetic polymorphisms in rs53576 and rs2254298	(Malhi et al., 2020; Womersley et al., 2020; Acosta et al., 2021)
	Human saliva	Negative association between brain volumes in maltreated children and OXTR DNA methylation	DNA methylation in OXTR gene	(Fujisawa et al., 2019)
Anxiety and depression	Human saliva	Higher DNA methylation was found in subjects with anxiety/depression	DNA methylation in OXTR gene	(Chagnon et al., 2015)
Others (social interactions, aggression, and, obsessive-compulsive disorder)	Human buccal mucosa cells	OXTR rs2254298 GG carriers showed fewer social interactions; rs53576 AA was associated with aggression	OXTR genetic polymorphisms in rs53576 and rs2254298	(Bonassi et al., 2020; Butovskaya et al., 2020)
	Human blood	Significant hypermethylation at CpG site cg04523291 in OCD patients	DNA methylation in cg04523291	(Bey et al., 2022)

### 6.1. ASD

Autism spectrum disorder (ASD) is an early-onset pervasive neurodevelopmental disorder that comprises a spectrum of cognitive and behavioral dysfunctions of childhood psychiatric disorder, with a population prevalence of about 1% and an estimated heritability of 80% worldwide (Ronald and Hoekstra, 2011; Lai et al., 2014). ASD is characterized by repetitive and stereotypical behavior, restricted interests, and impaired social interaction and communication, which has shown higher morbidity in male than female individuals (Ronald and Hoekstra, 2011; Lai et al., 2014). These profiles significantly affect neurodevelopment and maturity in childhood and even have lifelong consequences. Understanding the specific mechanisms underlying the neuropathogenesis of ASD remains difficult. Nevertheless, genetic and epigenetic evidence has been found to play pivotal roles in the etiology of ASD (Gregory et al., 2009; Wang et al., 2021).

Oxytocin receptor has been identified as a risk gene for ASD due to converging evidence from genetic and epigenetic analyses in ASD individuals (Gregory et al., 2009; LoParo and Waldman, 2015). Statistically significant hypermethylation of OXTR was found in the peripheral blood and temporal cortex tissue of autistic individuals compared to controls, as well as decreased OXTR mRNA expression in the temporal cortex tissue (Gregory et al., 2009). The correlation analysis showed that the decreased expression of the OXTR gene was related to the increased methylation in the temporal cortex (Gregory et al., 2009). In addition, Elissar Andari et al. found that ASD subjects showed higher hypermethylation in the intron 1 area of OXTR compared with neurotypical controls and that the hypermethylation in OXTR was correlated with the clinical severity of ASD, such as social responsiveness deficits and brain functional connectivity (Andari et al., 2020). Taken together, those findings provided substantial evidence for OXTR hypermethylation as a considerable risk biomarker of ASD.

Single-nucleotide polymorphisms (SNPs) are key genetic variations relevant to the development of a variety of neurodevelopmental disorders (Byrne et al., 2021). A study by Suping Wu et al. showed that two SNPs (rs2254298 and rs53576) located within the OXTR gene of 195 Chinese Han autism were significantly associated with ASD, suggesting an implication of OXTR in the susceptibility to ASD (Wu et al., 2005). Furthermore, a meta-analysis of 16 OXTR SNPs including 3,941 ASD patients from 11 independent samples was conducted to study whether variations in the OXTR gene were associated with ASD and to find out which particular SNPs showed a significant association with ASD (LoParo and Waldman, 2015). This study showed that there was a significant association between OXTR variations and ASD in a gene-based test and that several SNPs including rs2268491, rs7632287, rs237887, and rs2254298 were found to be significantly associated with ASD (LoParo and Waldman, 2015). Furthermore, a study by E Lerer et al. found that a five-locus haplotype block (rs237897-rs13316193-rs237889-rs2254298-rs2268494) showed a significant association with ASD (Lerer et al., 2008). These findings suggested that many different OXTR variations in SNPs were important and potential biomarkers of ASD.

Additionally, it was found that OXT had modulatory effects on social behaviors, including attachment, maternal behavior, mating, sexual behavior, and aggression, and showed significant improvements toward social deficits involved in psychiatric disorders, especially in ASD (Ferguson et al., 2001; Hoge et al., 2019; Froemke and Young, 2021). Hence, due to its pro-social effects, intranasal administration of OXT has been used for the treatment of adults and juveniles with ASD in clinical studies and has been considered a new therapeutic option for ASD (Anagnostou et al., 2012; Penagarikano, 2015; Parker et al., 2017), despite the dispute of benefit from OXT (Yamasue et al., 2020). Takamitsu Watanabe et al., found that the major allelotypes in two OXTR SNPs, including rs2254298 and rs53576, were correlated with the behavioral and neural responses to OXT treatment in ASD individuals, which suggested that the OXTR SNP variants had biological functions on autistic OXT efficacies (Watanabe et al., 2017). These findings implied that the OXTR SNP variants not only correlate with the development of ASD but also may function as predicted genetic biomarkers in clinical OXT responses before its actual treatment.

### 6.2. Borderline personality disorder

Borderline personality disorder (BPD) is a common psychiatric disorder characterized by a central feature of interpersonal dysfunction, which may be caused by a variety of biological, genetic, epigenetic, environmental factors, or gene–environment interactions. Accumulating evidence suggests that variation in gene polymorphism is a risk factor that may contribute to the disease phenotypes (Albert and Kruglyak, 2015). The sequencing of the human genome has made it possible to study the cause-and-effect relationship between genetic architecture and most of the common diseases. Common diseases are polygenic-related, with many loci contributing to the phenotype. The number of genomic variants or gene polymorphisms may contribute to the risk of disease (Visscher et al., 2021).

Genetic variations of the OXTR gene, including the most common SNPs, have been found in human psychiatric disorders. Recently, a clinical study among male inmates in China revealed that, compared with the non-BPD controls, BPD patients had higher frequencies in rs53576 AA and rs237987 AA genotypes, despite showing no statistical significance after Bonferroni correction (Zhang et al., 2020). The rs53576 GG genotype individuals with BPD showed higher BPD scores at higher levels of childhood maltreatment, including sexual abuse and physical abuse. These results indicate that the interaction between childhood maltreatment and genetic variations of OXTR makes a difference in the development of BPD. OXTR variations may be evidence for gene plasticity, functioning as important risk factors for BPD (Zhang et al., 2020). It was also reported that the AA-allelotype in OXTR rs53576 was linked to high levels of later BPD symptoms in youth with BPD (Hammen et al., 2015). Consequently, OXTR polymorphisms in rs53576 and rs237987 may serve as genetic markers and predictors of later symptoms in BPD.

### 6.3. ADHD

With an estimated world prevalence of about 5%, attention-deficit/hyperactivity disorder (ADHD) is one of the most frequent neurodevelopmental disorders in school-aged children who show substantial impairments in inattention, hyperactivity, impulsivity, and deficits in social cognitive abilities (Polanczyk et al., 2007; Cortese and Coghill, 2018). Although ADHD is considered a children's disease, impairing ADHD symptoms appear in adulthood in a considerable portion of cases (~65%) (Faraone et al., 2006; Cortese and Coghill, 2018). Recently, Tugba Kalyoncu et al. showed that the TT genotype in OXTR rs4686302 appeared to be more frequent in the children with ADHD, compared with that in the healthy controls, and that the ADHD children with the CT/TT genotype in OXTR rs4686302 displayed noticeably worse performance on a facial emotion recognition task than those with the CC genotype (Kalyoncu et al., 2019). These results might account for proof that different variations of the OXTR gene were correlated with different subtypes of children with ADHD. Additionally, similar research by J. Park et al. found that there was a significant association between SNP rs53576 of OXTR and social cognitive deficits in a subset of the ADHD probands, indicating that the AA genotype in OXTR rs53576 was correlated with better social cognitive ability compared to the AG genotype (Park et al., 2010). Although no significant association between the OXTR genotype and mRNA expression was found in this study, it confirmed previous findings that the OXTR gene was involved in social cognition (Park et al., 2010).

As one of the most common and important epigenetic modifications, DNA methylation status in specific sites, including exon and enhancer, regulates gene transcription and controls gene expression, directly or indirectly (Ehrlich and Lacey, 2013). Recently, it was reported that DNA methylation in the MT2 region of the OXTR gene, both in prairie voles and humans, was associated with an OXTR expression, suggesting that DNA methylation in specific sites of OXTR served as an indicator of the OXTR gene expression (Danoff et al., 2021). Michelle T. Siu et al. found that individuals with ADHD who exhibited extreme DNA methylation (DNAm) values in the OXTR gene displayed more social deficits and lower intelligence quotient (IQ), respectively, than those with normal DNAm levels. The abnormal DNAm status of OXTR was also uncovered in other neurodevelopmental disorders, especially in ASD (Siu et al., 2021), showing that OXTR DNAm patterns were altered in many psychiatric disorders.

### 6.4. Schizophrenia

Schizophrenia, a severe and heterogeneous psychiatric disorder, is characterized by impairments in social cognition, residual symptoms, and a chronic course in >50% of patients (Lambert et al., 2010). Under the fact that there are poor treatments for schizophrenia and limited research progress related to psychiatric disorders (DeLisi, 2005; Maric et al., 2016), the pathomechanism of schizophrenia is still unclear so far (Stepnicki et al., 2018). Recent studies have found that genetic polymorphisms in the OXTR and OXT genes in humans have been significantly related to schizophrenia and that OXTR and OXT gene variants have been involved in schizophrenia vulnerability and warrant independent replication (Souza et al., 2010; Teltsh et al., 2012; Montag et al., 2013). A case-control study showed that the OXTR SNPs rs53576 (A > G) and rs237885 (T > G) were significantly associated with a diagnosis indicator of schizophrenia and that OXTR SNPs rs53576 and rs237902 were significantly correlated with general psychopathology and negative symptom scores in schizophrenic patients, respectively (Montag et al., 2013). Furthermore, compared with healthy controls, schizophrenia patients showed downregulated expression of OXTR in the temporal cortex and decreased receptor binding in the vermis (Uhrig et al., 2016). The findings suggested that dysfunctions of OXTR including downregulation expression and genetic variants correlated with schizophrenia, which might contribute to the impairments of social cognition.

### 6.5. PTSD

Post-traumatic stress disorder (PTSD) is a severe mental disorder found in individuals who have directly experienced a traumatic event, such as traumatic re-experience symptoms, avoidance, numbness-like behaviors, and increased vigilance (Kirkpatrick and Heller, 2014). It has been reported that the lifelong prevalence of PTSD in adults is about 7.8% and that despite suffering fewer traumas, women show higher risk prevalence than men (Kessler et al., 2005; Ditlevsen and Elklit, 2012; Herringa, 2017). PTSD is characterized by aberrant function and structure in neural circuitry controlling emotion regulation and stress processing (Herringa, 2017). Although the cause that induces PTSD may be identifiable, the specific mechanism implicated in the structural and functional dysfunctions in the brain remains unclear. A recent study showed that the PTSD individuals who carried G allele in OXTR rs53576 had both noticeable PTSD-related symptoms and depressive symptoms (Cao et al., 2020). This study confirmed that the OXTR rs53576 genotype which was also found in other psychiatric disorders, such as ASD, BPD, ADHD, and Schizophrenia, was in connection with PTSD/depression comorbidity. Furthermore, it was found that female PTSD patients showed higher DNA methylation levels at two specific CpG islands (CpGs Chr3:8809437, Chr3:8809413) that were located at exon 3 of the OXTR, compared to PTSD males and female trauma-exposed controls (Nawijn et al., 2019). Moreover, within PTSD females, DNA methylation in the two CpG sites of OXTR showed a positive correlation with PTSD-related symptoms, including left amygdala responses to negative emotional faces and anhedonia symptoms. Accordingly, the above studies related to the interactions between the phenotypes and genetic variants or epigenetic modifications indicate that OXTR is a susceptibility risk gene to PTSD.

### 6.6. Alterations of brain volumes

With regard to its important roles in psychiatric disorders, OXTR has been investigated in other aspects related to abnormal behaviors and aberrant brain structure and function. In adolescents, OXTR SNP rs53576 might be related to hippocampal volumes, providing structural and functional support for social behaviors (Malhi et al., 2020). In the study, smaller left hippocampal volumes were found in the rs53576 AA carriers who experienced a higher emotional trauma, compared with those who suffered a minimal emotional trauma, but no differences in GG carriers, which suggested that the interactions between OXTR rs53576 and adversity were associated with the hippocampal volumes (Malhi et al., 2020). Similarly, rs2254298 A risk allele in OXTR was found to be associated with reduced left hippocampal volume, which was also shown to be independently relevant to reduce bilateral amygdalar volumes (Womersley et al., 2020). Furthermore, larger left hippocampal volumes were found in boys with the GG genotype of OXTR rs53576 (Acosta et al., 2021). A high level of OXTR methylation was negatively associated with gray matter volume in the left orbitofrontal cortex in children with maltreatment (Fujisawa et al., 2019). Those studies indicate that rs53576 AA and rs2254298 AA genotypes in OXTR are risk SNPs that may increase the susceptibilities to the effects of environmental factors on the brain structure, such as emotional trauma and childhood emotional neglect.

### 6.7. Others (social interactions, aggression, anxiety, and depression)

**Figure 5:**
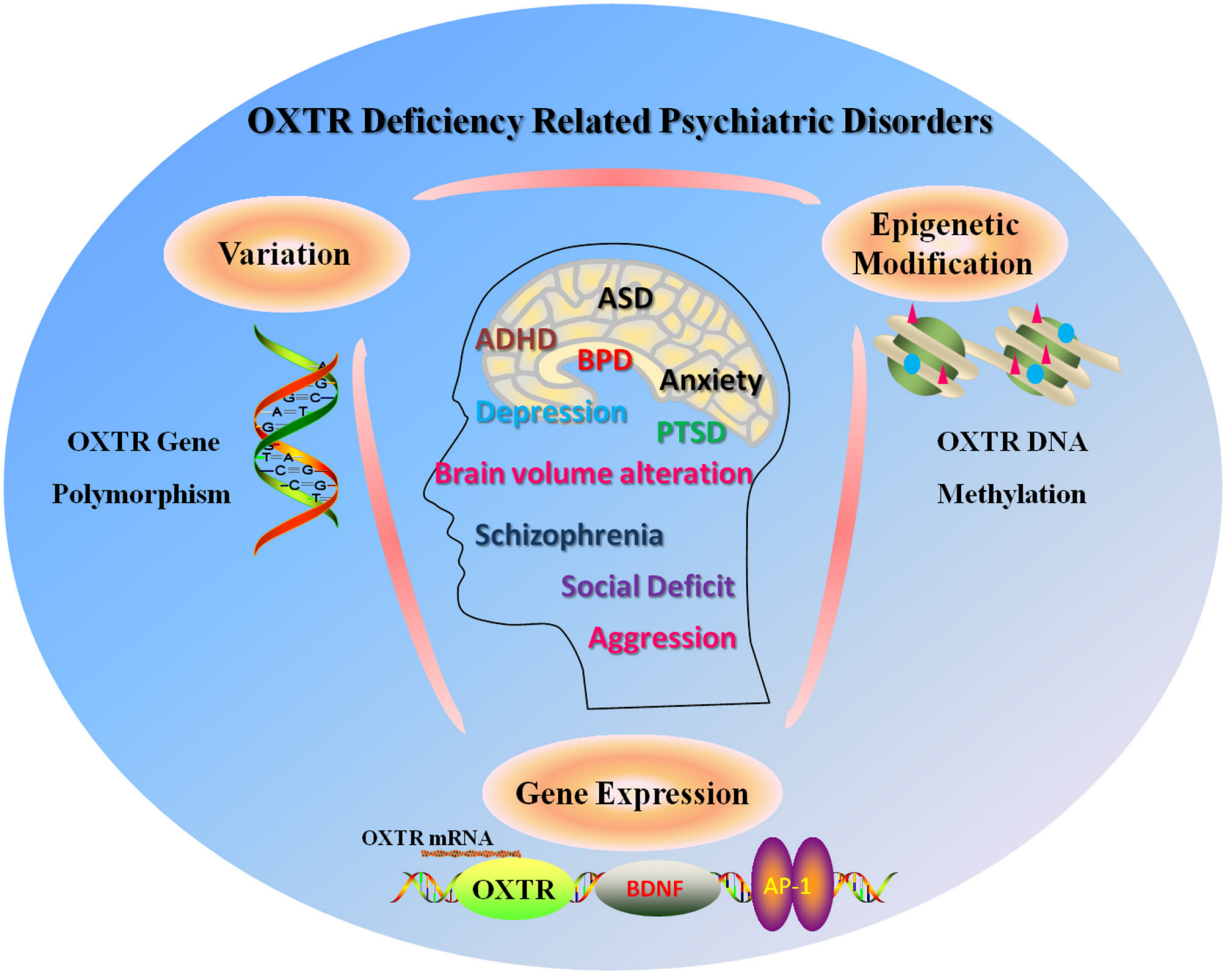
Involvement of OXTR variation, epigenetic modification, and gene expression in typical psychiatric disorders or alteration in brain development, including ASD, ADHD, BPD, PTSD, anxiety, depression, brain volume alteration, schizophrenia, and social deficit.

The individuals with the OXTR rs2254298 GG genotype and a history of low paternal care showed fewer social interactions than those with the AA genotype (Bonassi et al., 2020). These results showed that environmental factors interacted with gene variations to affect the phenotypes of psychiatric disorders and that OXTR was a susceptible risk gene to environmental factors. Additionally, a study on the association between OXTR polymorphisms and aggression found that the AA genotype in OXTR rs53576 in men served as a risk factor for aggression that was characterized by anger and hostility, suggesting that OXTR genetic polymorphism might affect emotional (anger) and cognitive (hostility) aggression in humans (Butovskaya et al., 2020). Epigenetic non-structural changes in the OXTR gene, such as DNA methylation that may mediate gene expression, are commonly found in psychiatric disorders (Siu et al., 2021). Obsessive-compulsive disorder (OCD) patients showed higher DNA methylation at the CpG site of cg04523291 of OXTR compared to controls (Schiele et al., 2021; Bey et al., 2022). A greater DNA methylation level in the OXTR gene was shown in subjects with anxiety and depression who carried the rs53576 AA genotype in the OXTR gene, in comparison with controls (Chagnon et al., 2015). Ludwig et al. found that OXTR promoter methylation level was positively but not significantly associated with the severity of depression symptoms in affective disorder individuals (Ludwig et al., 2022). Altogether, the evidence may support the roles of epigenetic and genetic changes in the OXTR gene in the development of many psychiatric disorders and the structural and functional abnormalities in the brain (Figure 5).

## 7. Summary and outlook

Overall, while some research questions involved in OXT/OXTR deficiency-related psychiatric disorders have been answered, many others remain to be addressed. The mechanisms underlying the dysregulation of OXT/OXTR signaling in neurodevelopmental disorders are not fully understood. Here we summarize the expression of OXTR and the involvement of OXT/OXTR signaling in the regulation of intracellular effects, behavioral responses, functional alterations, and the outcomes of OXTR dysregulation or deficiencies in behavioral deficits and psychiatric disorders. As reviewed above, the OXT/OXTR system regulates social cognition, social behaviors, emotions, and autonomic functions, including social memory, maternal behavior, attachment, mating, sexual behavior, depression, anxiety, aggression, and others. Due to the promising therapeutic outcomes in improving social recognition and behaviors, an intranasal OXT administration has been proposed to treat autism spectrum disorder. Whereas, recent studies have given rise to a contrary result that OXT shows no benefit for the youth with ASD. Thus, the clinical effectiveness of an intranasal OXT administration in patients with neurodevelopmental disorders remains to be investigated. Moreover, the intracellular effectors or downstream signaling of OXT/OXTR mainly include ERK/MAPK, eEF2 phosphorylation, NO production, PLCβ/PKC, KCC2 phosphorylation and expression, and the excitatory-to-inhibitory GABA switch, which may elucidate the intrinsic molecular mechanisms underlying the regulation of OXT/OXTR in social cognition and behaviors.

Due to the implications of OXTR in psychiatric disorders, more and more clinical studies have focused on the correlations between OXTR DNA sequence variations and phenotypes, and even the causality between individual polymorphisms and changes in gene expression, which in turn lead to physiological outcomes and, finally, disease risk. OXTR variations were implicated in ASD, ADHD, BPD, aggression, anxiety and depression, PTSD, schizophrenia, alterations of brain volumes, and other psychiatric disorders. The current studies, however, incompletely or partially accounted for the possibility that the variations, epigenetic modifications, or gene expressions in the OXTR gene might influence the development of diseases, and were limited to the clinical correlational investigation between abnormal OXT/OXTR signaling and psychiatric disorders. Taking into account the complexities and challenges in the field of psychiatric disorders that we are facing, additional studies need to be conducted for a better understanding of how the OXTR gene variations happen and how those variations affect the psychiatric phenotypes. The review summarizes the aberrant OXT/OXTR signaling in behavioral deficits and psychiatric disorders, contributing to the understanding of the neurobiology of psychiatric disorders and providing insights into the progress of OXTR-related neurodevelopmental disorders.

## Author contributions

JW and HZ: concept and design. GF and JW: literature search and manuscript drafting. HZ, GF, GL, ZC, and JY: critical revision. All authors contributed to the article and approved the submitted version.

## References

[B1] Acosta H. Tuulari J. J. Kantojarvi K. Lewis J. D. Hashempour N. Scheinin N. M.. (2021). A variation in the infant oxytocin receptor gene modulates infant hippocampal volumes in association with sex and prenatal maternal anxiety. Psychiatry Res. Neuroimaging 307, 111207. 10.1016/j.pscychresns.2020.111207 33168330

[B2] Albert F. W. Kruglyak L. (2015). The role of regulatory variation in complex traits and disease. Nat. Rev. Genet. 16, 197–212. 10.1038/nrg3891 25707927

[B3] Almeida D. Fiori L. M. Chen G. G. Aouabed Z. Lutz P. E. Zhang T. Y.. (2022). Oxytocin receptor expression and epigenetic regulation in the anterior cingulate cortex of individuals with a history of severe childhood abuse. Psychoneuroendocrinology 136, 105600. 10.1016/j.psyneuen.2021.105600 34839083

[B4] Anagnostou E. Soorya L. Chaplin W. Bartz J. Halpern D. Wasserman S.. (2012). Intranasal oxytocin vs. placebo in the treatment of adults with autism spectrum disorders: a randomized controlled trial. Mol. Autism 3, 16. 10.1186/2040-2392-3-16 23216716 PMC3539865

[B5] Andari E. Nishitani S. Kaundinya G. Caceres G. A. Morrier M. J. Ousley O.. (2020). Epigenetic modification of the oxytocin receptor gene: implications for autism symptom severity and brain functional connectivity. Neuropsychopharmacology 45, 1150–1158. 10.1038/s41386-020-0610-6 31931508 PMC7235273

[B6] Aoki Y. Yahata N. Watanabe T. Takano Y. Kawakubo Y. Kuwabara H.. (2014). Oxytocin improves behavioural and neural deficits in inferring others' social emotions in autism. Brain 137, 3073–3086. 10.1093/brain/awu231 25149412

[B7] Baker M. Lindell S. G. Driscoll C. A. Zhou Z. Yuan Q. Schwandt M. L.. (2017). Early rearing history influences oxytocin receptor epigenetic regulation in rhesus macaques. Proc. Natl. Acad. Sci. USA. 114, 11769–11774. 10.1073/pnas.1706206114 29078292 PMC5676889

[B8] Bartz J. A. Nitschke J. P. Krol S. A. Tellier P. P. (2019). Oxytocin selectively improves empathic accuracy: a replication in men and novel insights in women. Biol. Psychiatry Cogn. Neurosci. Neuroimaging 4, 1042–1048. 10.1016/j.bpsc.2019.01.014 30954442

[B9] Baumgartner T. Heinrichs M. Vonlanthen A. Fischbacher U. Fehr E. (2008). Oxytocin shapes the neural circuitry of trust and trust adaptation in humans. Neuron 58, 639–650. 10.1016/j.neuron.2008.04.009 18498743

[B10] Bey K. Campos-Martin R. Klawohn J. Reuter B. Grutzmann R. Riesel A.. (2022). Hypermethylation of the oxytocin receptor gene (OXTR). in obsessive-compulsive disorder: further evidence for a biomarker of disease and treatment response. Epigenetics 17, 642–652. 10.1080/15592294.2021.1943864 34269138 PMC9235899

[B11] Bonassi A. Cataldo I. Gabrieli G. Foo J. N. Lepri B. Esposito G.. (2020). Oxytocin receptor gene polymorphisms and early parental bonding interact in shaping instagram social behavior. Int. J. Environ. Res. Public Health 17, 7232. 10.3390/ijerph17197232 33022913 PMC7579356

[B12] Bos P. A. (2017). The endocrinology of human caregiving and its intergenerational transmission. Dev. Psychopathol. 29, 971–999. 10.1017/S0954579416000973 27760577

[B13] Braida D. Donzelli A. Martucci R. Capurro V. Busnelli M. Chini B.. (2012). Neurohypophyseal hormones manipulation modulate social and anxiety-related behavior in zebrafish. Psychopharmacology 220, 319–330. 10.1007/s00213-011-2482-2 21956239

[B14] Busnelli M. Chini B. (2018). Molecular basis of oxytocin receptor signalling in the brain: what we know and what we need to know. Curr. Top. Behav. Neurosci. 35, 3–29. 10.1007/7854_2017_6 28812263

[B15] Butovskaya M. Rostovtseva V. Butovskaya P. Burkova V. Dronova D. Filatova V.. (2020). Oxytocin receptor gene polymorphism (rs53576). and digit ratio associates with aggression: comparison in seven ethnic groups. J. Physiol. Anthropol. 39, 20. 10.1186/s40101-020-00232-y 32795360 PMC7427763

[B16] Byrne E. M. Zhu Z. Qi T. Skene N. G. Bryois J. Pardinas A. F.. (2021). Conditional GWAS analysis to identify disorder-specific SNPs for psychiatric disorders. Mol. Psychiatry 26, 2070–2081. 10.1038/s41380-020-0705-9 32398722 PMC7657979

[B17] Cao C. Wang L. Wu J. Li G. Fang R. Liu P.. (2020). Association between the OXTR rs53576 genotype and latent profiles of post-traumatic stress disorder and depression symptoms in a representative sample of earthquake survivors. Anxiety Stress Coping 33, 140–147. 10.1080/10615806.2019.1695604 31771350

[B18] Carter C. S. (2014). Oxytocin pathways and the evolution of human behavior. Annu. Rev. Psychol. 65, 17–39. 10.1146/annurev-psych-010213-115110 24050183

[B19] Cataldo I. Azhari A. Lepri B. Esposito G. (2018). Oxytocin receptors (OXTR). and early parental care: an interaction that modulates psychiatric disorders. Res. Dev. Disabil. 82, 27–38. 10.1016/j.ridd.2017.10.007 29033100

[B20] Cater M. Majdic G. (2022). How early maternal deprivation changes the brain and behavior? Eur. J. Neurosci. 55, 2058–2075. 10.1111/ejn.15238 33870558

[B21] Cattaneo M. G. Chini B. Vicentini L. M. (2008). Oxytocin stimulates migration and invasion in human endothelial cells. Br. J. Pharmacol. 153, 728–736. 10.1038/sj.bjp.0707609 18059319 PMC2259201

[B22] Chagnon Y. C. Potvin O. Hudon C. Preville M. (2015). DNA methylation and single nucleotide variants in the brain-derived neurotrophic factor (BDNF) and oxytocin receptor (OXTR) genes are associated with anxiety/depression in older women. Front. Genet. 6, 230. 10.3389/fgene.2015.00230 26175754 PMC4485183

[B23] Chang S. W. Platt M. L. (2014). Oxytocin and social cognition in rhesus macaques: implications for understanding and treating human psychopathology. Brain Res. 1580, 57–68. 10.1016/j.brainres.2013.11.006 24231551 PMC4017005

[B24] Cherepanov S. M. Gerasimenko M. Yuhi T. Furuhara K. Tsuji C. Yokoyama S.. (2021). Oxytocin ameliorates impaired social behavior in a Chd8 haploinsufficiency mouse model of autism. BMC Neurosci. 22, 32. 10.1186/s12868-021-00631-6 33933000 PMC8088024

[B25] Chiang M. C. Huang A. J. Y. Wintzer M. E. Ohshima T. McHugh T. J. (2018). A role for CA3 in social recognition memory. Behav. Brain Res. 354, 22–30. 10.1016/j.bbr.2018.01.019 29355673

[B26] Cho S. Y. Kim A. Y. Kim J. Choi D. H. Son E. D. Shin D. W.. (2019). Oxytocin alleviates cellular senescence through oxytocin receptor-mediated extracellular signal-regulated kinase/Nrf2 signalling. Br. J. Dermatol. 181, 1216–1225. 10.1111/bjd.17824 30801661

[B27] Choe K. Y. Bethlehem R. A. I. Safrin M. Dong H. Salman E. Li Y.. (2022). Oxytocin normalizes altered circuit connectivity for social rescue of the Cntnap2 knockout mouse. Neuron 110, 795–808. e796. 10.1016/j.neuron.2021.11.031 34932941 PMC8944915

[B28] Cortese S. Coghill D. (2018). Twenty years of research on attention-deficit/hyperactivity disorder (ADHD): looking back, looking forward. Evid. Based Ment. Health 21, 173–176. 10.1136/ebmental-2018-300050 30301823 PMC10270437

[B29] Dadds M. R. MacDonald E. Cauchi A. Williams K. Levy F. Brennan J.. (2014). Nasal oxytocin for social deficits in childhood autism: a randomized controlled trial. J. Autism Dev. Disord. 44, 521–531. 10.1007/s10803-013-1899-3 23888359

[B30] Danoff J. S. Wroblewski K. L. Graves A. J. Quinn G. C. Perkeybile A. M. Kenkel W. M.. (2021). Genetic, epigenetic, and environmental factors controlling oxytocin receptor gene expression. Clin. Epigenetics 13, 23. 10.1186/s13148-021-01017-5 33516250 PMC7847178

[B31] De Dreu C. K. Greer L. L. Handgraaf M. J. Shalvi S. Van Kleef G. A. Baas M.. (2010). The neuropeptide oxytocin regulates parochial altruism in intergroup conflict among humans. Science. 328, 1408–1411. 10.1126/science.1189047 20538951

[B32] DeLisi L. E. (2005). Current concepts in schizophrenia research: advancing progress toward understanding etiology and new treatments in year 2004. Curr. Opin. Psychiatry 18, 109–110. 10.1097/00001504-200503000-00002 16639162

[B33] Devost D. Wrzal P. Zingg H. H. (2008). Oxytocin receptor signalling. Prog. Brain Res. 170, 167–176. 10.1016/S0079-6123(08)00415-9 18655881

[B34] Di Simplicio M. Massey-Chase R. Cowen P. J. Harmer C. J. (2009). Oxytocin enhances processing of positive vs. negative emotional information in healthy male volunteers. J. Psychopharmacol. 23, 241–248. 10.1177/0269881108095705 18801829

[B35] Ditlevsen D. N. Elklit A. (2012). Gender, trauma type, and PTSD prevalence: a re-analysis of 18 nordic convenience samples. Ann. Gen. Psychiatry 11, 26. 10.1186/1744-859X-11-26 23107002 PMC3494556

[B36] Ditzen B. Schaer M. Gabriel B. Bodenmann G. Ehlert U. Heinrichs M. (2009). Intranasal oxytocin increases positive communication and reduces cortisol levels during couple conflict. Biol. Psychiatry 65, 728–731. 10.1016/j.biopsych.2008.10.011 19027101

[B37] Domes G. Lischke A. Berger C. Grossmann A. Hauenstein K. Heinrichs M.. (2010). Effects of intranasal oxytocin on emotional face processing in women. Psychoneuroendocrinology 35, 83–93. 10.1016/j.psyneuen.2009.06.016 19632787

[B38] Eftekhari S. Shahrokhi A. Tsintsadze V. Nardou R. Brouchoud C. Conesa M.. (2014). Response to Comment on “Oxytocin-mediated GABA inhibition during delivery attenuates autism pathogenesis in rodent offspring”. Science. 346, 176. 10.1126/science.1256009 25301611

[B39] Ehrlich M. Lacey M. (2013). DNA methylation and differentiation: silencing, upregulation and modulation of gene expression. Epigenomics 5, 553–568. 10.2217/epi.13.43 24059801 PMC3864898

[B40] Faraone S. V. Biederman J. Mick E. (2006). The age-dependent decline of attention deficit hyperactivity disorder: a meta-analysis of follow-up studies. Psychol. Med. 36, 159–165. 10.1017/S003329170500471X 16420712

[B41] Ferguson J. N. Aldag J. M. Insel T. R. Young L. J. (2001). Oxytocin in the medial amygdala is essential for social recognition in the mouse. J. Neurosci. 21, 8278–8285. 10.1523/JNEUROSCI.21-20-08278.2001 11588199 PMC6763861

[B42] Fischer-Shofty M. Shamay-Tsoory S. G. Levkovitz Y (2013). Characterization of the effects of oxytocin on fear recognition in patients with schizophrenia and in healthy controls. Front. Neurosci. 7, 127. 10.3389/fnins.2013.00127 23882178 PMC3714571

[B43] Fitts D. A. Thornton S. N. Ruhf A. A. Zierath D. K. Johnson A. K. Thunhorst R. L. (2003). Effects of central oxytocin receptor blockade on water and saline intake, mean arterial pressure, and c-Fos expression in rats. Am. J. Physiol. Regul. Integr. Comp. Physiol. 285, R1331–1339. 10.1152/ajpregu.00254.2003 12907413

[B44] Froemke R. C. Young L. J. (2021). Oxytocin, neural plasticity, and social behavior. Annu. Rev. Neurosci. 44, 359–381. 10.1146/annurev-neuro-102320-102847 33823654 PMC8604207

[B45] Fujisawa T. X. Nishitani S. Takiguchi S. Shimada K. Smith A. K. Tomoda A. (2019). Oxytocin receptor DNA methylation and alterations of brain volumes in maltreated children. Neuropsychopharmacology 44, 2045–2053. 10.1038/s41386-019-0414-8 31071720 PMC6898679

[B46] Gong L. Gao F. Li J. Li J. Yu X. Ma X.. (2015). Oxytocin-induced membrane hyperpolarization in pain-sensitive dorsal root ganglia neurons mediated by Ca(2+)/nNOS/NO/KATP pathway. Neuroscience 289, 417–428. 10.1016/j.neuroscience.2014.12.058 25617653

[B47] Gregory S. G. Connelly J. J. Towers A. J. Johnson J. Biscocho D. Markunas C. A.. (2009). Genomic and epigenetic evidence for oxytocin receptor deficiency in autism. BMC Med. 7, 62. 10.1186/1741-7015-7-62 19845972 PMC2774338

[B48] Guastella A. J. Einfeld S. L. Gray K. M. Rinehart N. J. Tonge B. J. Lambert T. J.. (2010). Intranasal oxytocin improves emotion recognition for youth with autism spectrum disorders. Biol. Psychiatry 67, 692–694. 10.1016/j.biopsych.2009.09.020 19897177

[B49] Guastella A. J. Gray K. M. Rinehart N. J. Alvares G. A. Tonge B. J. Hickie I. B.. (2015a). The effects of a course of intranasal oxytocin on social behaviors in youth diagnosed with autism spectrum disorders: a randomized controlled trial. J. Child Psychol. Psychiatry 56, 444–452. 10.1111/jcpp.12305 25087908

[B50] Guastella A. J. Mitchell P. B. Dadds M. R. (2008a). Oxytocin increases gaze to the eye region of human faces. Biol. Psychiatry 63, 3–5. 10.1016/j.biopsych.2007.06.026 17888410

[B51] Guastella A. J. Mitchell P. B. Mathews F. (2008b). Oxytocin enhances the encoding of positive social memories in humans. Biol. Psychiatry 64, 256–258. 10.1016/j.biopsych.2008.02.008 18343353

[B52] Guastella A. J. Ward P. B. Hickie I. B. Shahrestani S. Hodge M. A. Scott E. M.. (2015b). A single dose of oxytocin nasal spray improves higher-order social cognition in schizophrenia. Schizophr. Res. 168, 628–633. 10.1016/j.schres.2015.06.005 26150070

[B53] Hammen C. Bower J. E. Cole S. W. (2015). Oxytocin receptor gene variation and differential susceptibility to family environment in predicting youth borderline symptoms. J. Pers. Disord. 29, 177–192. 10.1521/pedi_2014_28_152 25102084

[B54] Harony-Nicolas H. Kay M. du Hoffmann J. Klein M. E. Bozdagi-Gunal O. Riad M.. (2017). Oxytocin improves behavioral and electrophysiological deficits in a novel Shank3-deficient rat. Elife 6, e18904. 10.7554/eLife.18904 28139198 PMC5283828

[B55] Heise C. Taha E. Murru L. Ponzoni L. Cattaneo A. Guarnieri F. C.. (2017). eEF2K/eEF2 Pathway controls the excitation/inhibition balance and susceptibility to epileptic seizures. Cereb. Cortex 27, 2226–2248. 10.1093/cercor/bhw075 27005990 PMC5963824

[B56] Herringa R. J. (2017). Trauma, PTSD, and the developing brain. Curr. Psychiatry Rep. 19, 69. 10.1007/s11920-017-0825-3 28823091 PMC5604756

[B57] Hoge E. Bui E. Rosencrans P. Orr S. Ross R. Ojserkis R.. (2019). Influence of intranasal oxytocin on fear consolidation in healthy humans. Gen. Psychiatr. 32, e100131. 10.1136/gpsych-2019-100131 31922086 PMC6936973

[B58] Inoue T. Kimura T. Azuma C. Inazawa J. Takemura M. Kikuchi T.. (1994). Structural organization of the human oxytocin receptor gene. J. Biol. Chem. 269, 32451–32456. 10.1016/S0021-9258(18)31656-9 7798245

[B59] Iroegbu J. D. Ijomone O. K. Femi-Akinlosotu O. M. Ijomone O. M. (2021). ERK/MAPK signalling in the developing brain: perturbations and consequences. Neurosci. Biobehav. Rev. 131, 792–805. 10.1016/j.neubiorev.2021.10.009 34634357

[B60] Ji L. Chen C. Hou B. Ren D. Yuan F. Liu L.. (2021). Impact of OXTR Polymorphisms on subjective wellbeing: the intermediary role of attributional style. Front. Genet. 12, 763628. 10.3389/fgene.2021.763628 35222513 PMC8864163

[B61] Jones C. Barrera I. Brothers S. Ring R. Wahlestedt C. (2017). Oxytocin and social functioning. Dialogues Clin. Neurosci. 19, 193–201. 10.31887/DCNS.2017.19.2/cjones 28867943 PMC5573563

[B62] Jurek B. Neumann I. D. (2018). The oxytocin receptor: from intracellular signaling to behavior. Physiol. Rev. 98, 1805–1908. 10.1152/physrev.00031.2017 29897293

[B63] Jurek B. Slattery D. A. Hiraoka Y. Liu Y. Nishimori K. Aguilera G.. (2015). Oxytocin regulates stress-induced crf gene transcription through creb-regulated transcription coactivator 3. J. Neurosci. 35, 12248–12260. 10.1523/JNEUROSCI.1345-14.2015 26338335 PMC4556790

[B64] Kahle K. T. Khanna A. R. Alper S. L. Adragna N. C. Lauf P. K. Sun D.. (2015). K-Cl cotransporters, cell volume homeostasis, and neurological disease. Trends Mol. Med. 21, 513–523. 10.1016/j.molmed.2015.05.008 26142773 PMC4834970

[B65] Kalyoncu T. Ozbaran B. Kose S. Onay H. (2019). Variation in the oxytocin receptor gene is associated with social cognition and ADHD. J. Atten. Disord. 23, 702–711. 10.1177/1087054717706757 28478728

[B66] Kelly A. M. Wilson L. C. (2020). Aggression: perspectives from social and systems neuroscience. Horm. Behav. 123, 104523. 10.1016/j.yhbeh.2019.04.010 31002771

[B67] Kendrick K. M. Guastella A. J. Becker B. (2018). Overview of human oxytocin research. Curr. Top. Behav. Neurosci. 35, 321–348. 10.1007/7854_2017_19 28864976

[B68] Keri S. Benedek G. (2009). Oxytocin enhances the perception of biological motion in humans. Cogn. Affect. Behav. Neurosci. 9, 237–241. 10.3758/CABN.9.3.237 19679759

[B69] Kessler R. C. Berglund P. Demler O. Jin R. Merikangas K. R. Walters E. E. (2005). Lifetime prevalence and age-of-onset distributions of DSM-IV disorders in the National comorbidity survey replication. Arch. Gen. Psychiatry 62, 593–602. 10.1001/archpsyc.62.6.593 15939837

[B70] Kim E. K. Choi E. J. (2010). Pathological roles of MAPK signaling pathways in human diseases. Biochim. Biophys. Acta 1802, 396–405. 10.1016/j.bbadis.2009.12.009 20079433

[B71] Kimura R. Tomiwa K. Inoue R. Suzuki S. Nakata M. Awaya T.. (2020). Dysregulation of the oxytocin receptor gene in Williams syndrome. Psychoneuroendocrinology 115, 104631. 10.1016/j.psyneuen.2020.104631 32114409

[B72] Kimura T. Saji F. Nishimori K. Ogita K. Nakamura H. Koyama M.. (2003). Molecular regulation of the oxytocin receptor in peripheral organs. J. Mol. Endocrinol. 30, 109–115. 10.1677/jme.0.0300109 12683935

[B73] Kimura T. Tanizawa O. Mori K. Brownstein M. J. Okayama H. (1992). Structure and expression of a human oxytocin receptor. Nature 356, 526–529. 10.1038/356526a0 1313946

[B74] Kirkpatrick H. A. Heller G. M. (2014). Post-traumatic stress disorder: theory and treatment update. Int. J. Psychiatry Med. 47, 337–346. 10.2190/PM.47.4.h 25084856

[B75] Kitagawa K. Matsumura K. Baba M. Kondo M. Takemoto T. Nagayasu K.. (2021). Intranasal oxytocin administration ameliorates social behavioral deficits in a POGZ(WT/Q1038R). mouse model of autism spectrum disorder. Mol. Brain 14, 56. 10.1186/s13041-021-00769-8 33726803 PMC7962304

[B76] Kosfeld M. Heinrichs M. Zak P. J. Fischbacher U. Fehr E (2005). Oxytocin increases trust in humans. Nature 435, 673–676. 10.1038/nature03701 15931222

[B77] Kusui C. Kimura T. Ogita K. Nakamura H. Matsumura Y. Koyama M.. (2001). DNA methylation of the human oxytocin receptor gene promoter regulates tissue-specific gene suppression. Biochem. Biophys. Res. Commun. 289, 681–686. 10.1006/bbrc.2001.6024 11726201

[B78] Lai M. C. Lombardo M. V. Baron-Cohen S. (2014). Autism. Lancet 383, 896–910. 10.1016/S0140-6736(13)61539-1 24074734

[B79] Lambert M. Karow A. Leucht S. Schimmelmann B. G. Naber D. (2010). Remission in schizophrenia: validity, frequency, predictors, and patients' perspective 5 years later. Dialogues Clin. Neurosci. 12, 393–407. 10.31887/DCNS.2010.12.3/mlambert 20954433 PMC3181974

[B80] Leonzino M. Busnelli M. Antonucci F. Verderio C. Mazzanti M. Chini B. (2016). The Timing of the excitatory-to-inhibitory GABA switch is regulated by the oxytocin receptor *via* KCC2. Cell Rep. 15, 96–103. 10.1016/j.celrep.2016.03.013 27052180 PMC4826440

[B81] Lerer E. Levi S. Salomon S. Darvasi A. Yirmiya N. Ebstein R. P. (2008). Association between the oxytocin receptor (OXTR). gene and autism: relationship to Vineland Adaptive Behavior Scales and cognition. Mol. Psychiatry 13, 980–988. 10.1038/sj.mp.4002087 17893705

[B82] Lin S. H. Kiyohara T. Sun B. (2003). Maternal behavior: activation of the central oxytocin receptor system in parturient rats? Neuroreport 14, 1439–1444. 10.1097/00001756-200308060-00007 12960760

[B83] Lin Y. T. Hsieh T. Y. Tsai T. C. Chen C. C. Huang C. C. Hsu K. S. (2018). Conditional deletion of hippocampal CA2/CA3a oxytocin receptors impairs the persistence of long-term social recognition memory in mice. J. Neurosci. 38, 1218–1231. 10.1523/JNEUROSCI.1896-17.2017 29279308 PMC6596267

[B84] Liu J. Liang Y. Jiang X. Xu J. Sun Y. Wang Z.. (2021). Maternal diabetes-induced suppression of oxytocin receptor contributes to social deficits in offspring. Front. Neurosci. 15, 634781. 10.3389/fnins.2021.634781 33633538 PMC7900564

[B85] LoParo D. Waldman I. D. (2015). The oxytocin receptor gene (OXTR). is associated with autism spectrum disorder: a meta-analysis. Mol. Psychiatry 20, 640–646. 10.1038/mp.2014.77 25092245

[B86] Ludwig B. Carlberg L. Kienesberger K. Swoboda P. Swoboda M. M. M. Bernegger A.. (2022). Oxytocin receptor gene methylation as a molecular marker for severity of depressive symptoms in affective disorder patients. BMC Psychiatry 22, 381. 10.1186/s12888-022-04031-w 35672748 PMC9172116

[B87] Lukas M. Toth I. Veenema A. H. Neumann I. D. (2013). Oxytocin mediates rodent social memory within the lateral septum and the medial amygdala depending on the relevance of the social stimulus: male juvenile vs. female adult conspecifics. Psychoneuroendocrinology 38, 916–926. 10.1016/j.psyneuen.2012.09.018 23102690

[B88] Malhi G. S. Das P. Outhred T. Dobson-Stone C. Bell E. Gessler D.. (2020). Interactions of OXTR rs53576 and emotional trauma on hippocampal volumes and perceived social support in adolescent girls. Psychoneuroendocrinology 115, 104635. 10.1016/j.psyneuen.2020.104635 32199286

[B89] Maric N. P. Jovicic M. J. Mihaljevic M. Miljevic C. (2016). Improving current treatments for Schizophrenia. Drug Dev. Res. 77, 357–367. 10.1002/ddr.21337 27633376

[B90] Melis M. R. Succu S. Iannucci U. Argiolas A. (1997). Oxytocin increases nitric oxide production in the paraventricular nucleus of the hypothalamus of male rats: correlation with penile erection and yawning. Regul. Pept. 69, 105–111. 10.1016/S0167-0115(97)00002-5 9178353

[B91] Montag C. Brockmann E. M. Bayerl M. Rujescu D. Muller D. J. Gallinat J. (2013). Oxytocin and oxytocin receptor gene polymorphisms and risk for schizophrenia: a case-control study. World J. Biol. Psychiatry 14, 500–508. 10.3109/15622975.2012.677547 22651577

[B92] Nawijn L. Krzyzewska I. M. van Zuiden M. Henneman P. Koch S. B. J. Mul A. N.. (2019). Oxytocin receptor gene methylation in male and female PTSD patients and trauma-exposed controls. Eur. Neuropsychopharmacol. 29, 147–155. 10.1016/j.euroneuro.2018.10.006 30415783

[B93] Neumann I. D. Slattery D. A. (2016). Oxytocin in general anxiety and social fear: a translational approach. Biol. Psychiatry 79, 213–221. 10.1016/j.biopsych.2015.06.004 26208744

[B94] Pagani J. H. Williams Avram S. K. Cui Z. Song J. Mezey E. Senerth J. M.. (2015). Raphe serotonin neuron-specific oxytocin receptor knockout reduces aggression without affecting anxiety-like behavior in male mice only. Genes Brain Behav. 14, 167–176. 10.1111/gbb.12202 25677455 PMC4536906

[B95] Park J. Willmott M. Vetuz G. Toye C. Kirley A. Hawi Z.. (2010). Evidence that genetic variation in the oxytocin receptor (OXTR). gene influences social cognition in ADHD. Prog. Neuropsychopharmacol. Biol. Psychiatry 34, 697–702. 10.1016/j.pnpbp.2010.03.029 20347913

[B96] Parker K. J. Oztan O. Libove R. A. Sumiyoshi R. D. Jackson L. P. Karhson D. S.. (2017). Intranasal oxytocin treatment for social deficits and biomarkers of response in children with autism. Proc. Natl. Acad. Sci. USA. 114, 8119–8124. 10.1073/pnas.1705521114 28696286 PMC5544319

[B97] Penagarikano O. (2015). New therapeutic options for autism spectrum disorder: experimental evidences. Exp. Neurobiol. 24, 301–311. 10.5607/en.2015.24.4.301 26713078 PMC4688330

[B98] Pobbe R. L. Pearson B. L. Defensor E. B. Bolivar V. J. Young W. S. Lee H. J.. (2012). Oxytocin receptor knockout mice display deficits in the expression of autism-related behaviors. Horm. Behav. 61, 436–444. 10.1016/j.yhbeh.2011.10.010 22100185 PMC3373312

[B99] Polanczyk G. De Lima M. S. Horta B. L. Biederman J. Rohde L. A. (2007). The worldwide prevalence of ADHD: a systematic review and metaregression analysis. Am. J. Psychiatry 164, 942–948. 10.1176/ajp.2007.164.6.942 17541055

[B100] Rae M. Lemos Duarte M. Gomes I. Camarini R. Devi L. A. (2022). Oxytocin and vasopressin: signalling, behavioural modulation and potential therapeutic effects. Br. J. Pharmacol. 179, 1544–1564. 10.1111/bph.15481 33817785 PMC8488062

[B101] Ramo-Fernandez L. Gumpp A. M. Boeck C. Krause S. Bach A. M. Waller C.. (2021). Associations between childhood maltreatment and DNA methylation of the oxytocin receptor gene in immune cells of mother-newborn dyads. Transl. Psychiatry 11, 449. 10.1038/s41398-021-01546-w 34471100 PMC8410844

[B102] Reichova A. Bacova Z. Bukatova S. Kokavcova M. Meliskova V. Frimmel K.. (2020). Abnormal neuronal morphology and altered synaptic proteins are restored by oxytocin in autism-related SHANK3 deficient model. Mol. Cell. Endocrinol. 518, 110924. 10.1016/j.mce.2020.110924 32619581

[B103] Rich M. E. deCardenas E. J. Lee H. J. Caldwell H. K. (2014). Impairments in the initiation of maternal behavior in oxytocin receptor knockout mice. PLoS ONE 9, e98839. 10.1371/journal.pone.0098839 24892749 PMC4044031

[B104] Ring R. H. Malberg J. E. Potestio L. Ping J. Boikess S. Luo B.. (2006). Anxiolytic-like activity of oxytocin in male mice: behavioral and autonomic evidence, therapeutic implications. Psychopharmacology 185, 218–225. 10.1007/s00213-005-0293-z 16418825

[B105] Ronald A. Hoekstra R. A. (2011). Autism spectrum disorders and autistic traits: a decade of new twin studies. Am. J. Med. Genet. B Neuropsychiatr. Genet. 156B, 255–74. 10.1002/ajmg.b.31159 21438136

[B106] Sack M. Spieler D. Wizelman L. Epple G. Stich J. Zaba M.. (2017). Intranasal oxytocin reduces provoked symptoms in female patients with posttraumatic stress disorder despite exerting sympathomimetic and positive chronotropic effects in a randomized controlled trial. BMC Med. 15, 40. 10.1186/s12916-017-0801-0 28209155 PMC5314583

[B107] Sala M. Braida D. Donzelli A. Martucci R. Busnelli M. Bulgheroni E.. (2013). Mice heterozygous for the oxytocin receptor gene (Oxtr(+/-)). show impaired social behaviour but not increased aggression or cognitive inflexibility: evidence of a selective haploinsufficiency gene effect. J. Neuroendocrinol. 25, 107–118. 10.1111/j.1365-2826.2012.02385.x 22967062

[B108] Sala M. Braida D. Lentini D. Busnelli M. Bulgheroni E. Capurro V.. (2011). Pharmacologic rescue of impaired cognitive flexibility, social deficits, increased aggression, and seizure susceptibility in oxytocin receptor null mice: a neurobehavioral model of autism. Biol. Psychiatry 69, 875–882. 10.1016/j.biopsych.2010.12.022 21306704

[B109] Satoh Y. Endo S. Nakata T. Kobayashi Y. Yamada K. Ikeda T.. (2011). ERK2 contributes to the control of social behaviors in mice. J. Neurosci. 31, 11953–11967. 10.1523/JNEUROSCI.2349-11.2011 21849556 PMC6623182

[B110] Schiele M. A. Thiel C. Kollert L. Furst L. Putschin L. Kehle R.. (2021). Oxytocin Receptor gene DNA methylation: a biomarker of treatment response in obsessive-compulsive disorder? Psychother. Psychosom. 90, 57–63. 10.1159/000509910 32920561

[B111] Sikich L. Kolevzon A. King B. H. McDougle C. J. Sanders K. B. Kim S. J.. (2021). Intranasal oxytocin in children and adolescents with autism spectrum disorder. N. Engl. J. Med. 385, 1462–1473. 10.1056/NEJMoa2103583 34644471 PMC9701092

[B112] Simmons C. F. Clancy T. E. Quan R. Knoll J. H. (1995). The oxytocin receptor gene (OXTR). localizes to human chromosome 3p25 by fluorescence in situ hybridization and PCR analysis of somatic cell hybrids. Genomics 26, 623–625. 10.1016/0888-7543(95)80188-R 7607693

[B113] Siu M. T. Goodman S. J. Yellan I. Butcher D. T. Jangjoo M. Grafodatskaya D.. (2021). DNA methylation of the oxytocin receptor across neurodevelopmental disorders. J Autism Dev Disord. 51, 3610–3623. 10.1007/s10803-020-04792-x 33394241

[B114] Souza R. P. Ismail P. Meltzer H. Y. Kennedy J. L. (2010). Variants in the oxytocin gene and risk for schizophrenia. Schizophr. Res. 121, 279–280. 10.1016/j.schres.2010.04.019 20547038

[B115] Steinert J. R. Chernova T. Forsythe I. D. (2010). Nitric oxide signaling in brain function, dysfunction, and dementia. Neuroscientist 16, 435–452. 10.1177/1073858410366481 20817920

[B116] Stepnicki P. Kondej M. Kaczor A. A. (2018). Current concepts and treatments of schizophrenia. Molecules 23, 2087. 10.3390/molecules23082087 30127324 PMC6222385

[B117] Sue Carter C. (2018). Oxytocin and human evolution. Curr. Top. Behav. Neurosci. 35, 291–319. 10.1007/7854_2017_18 28812268

[B118] Sun J. Nan G. (2016). The mitogen-activated protein kinase (MAPK). signaling pathway as a discovery target in stroke. J. Mol. Neurosci. 59, 90–98. 10.1007/s12031-016-0717-8 26842916

[B119] Sun Y. Liu W. Z. Liu T. Feng X. Yang N. Zhou H. F. (2015). Signaling pathway of MAPK/ERK in cell proliferation, differentiation, migration, senescence and apoptosis. J. Recept. Signal Transduct. Res. 35, 600–604. 10.3109/10799893.2015.1030412 26096166

[B120] Suraev A. S. Bowen M. T. Ali S. O. Hicks C. Ramos L. McGregor I. S. (2014). Adolescent exposure to oxytocin, but not the selective oxytocin receptor agonist TGOT, increases social behavior and plasma oxytocin in adulthood. Horm. Behav. 65, 488–496. 10.1016/j.yhbeh.2014.03.002 24631584

[B121] Takayanagi Y. Yoshida M. Bielsky I. F. Ross H. E. Kawamata M. Onaka T.. (2005). Pervasive social deficits, but normal parturition, in oxytocin receptor-deficient mice. Proc. Natl. Acad. Sci. USA. 102, 16096–16101. 10.1073/pnas.0505312102 16249339 PMC1276060

[B122] Tan Y. Singhal S. M. Harden S. W. Cahill K. M. Nguyen D. M. Colon-Perez L. M.. (2019). Oxytocin receptors are expressed by glutamatergic prefrontal cortical neurons that selectively modulate social recognition. J. Neurosci. 39, 3249–3263. 10.1523/JNEUROSCI.2944-18.2019 30804095 PMC6788819

[B123] Tang X. Jaenisch R. Sur M. (2021). The role of GABAergic signalling in neurodevelopmental disorders. Nat. Rev. Neurosci. 22, 290–307. 10.1038/s41583-021-00443-x 33772226 PMC9001156

[B124] Teltsh O. Kanyas-Sarner K. Rigbi A. Greenbaum L. Lerer B. Kohn Y. (2012). Oxytocin and vasopressin genes are significantly associated with schizophrenia in a large Arab-Israeli pedigree. Int. J. Neuropsychopharmacol. 15, 309–319. 10.1017/S1461145711001374 21899794

[B125] Theodoridou A. Rowe A. C. Penton-Voak I. S. Rogers P. J. (2009). Oxytocin and social perception: oxytocin increases perceived facial trustworthiness and attractiveness. Horm. Behav. 56, 128–132. 10.1016/j.yhbeh.2009.03.019 19344725

[B126] Tomizawa K. Iga N. Lu Y. F. Moriwaki A. Matsushita M. Li S. T.. (2003). Oxytocin improves long-lasting spatial memory during motherhood through MAP kinase cascade. Nat. Neurosci. 6, 384–390. 10.1038/nn1023 12598900

[B127] Tyzio R. Nardou R. Ferrari D. C. Tsintsadze T. Shahrokhi A. Eftekhari S.. (2014). Oxytocin-mediated GABA inhibition during delivery attenuates autism pathogenesis in rodent offspring. Science 343, 675–679. 10.1126/science.1247190 24503856

[B128] Uhrig S. Hirth N. Broccoli L. von Wilmsdorff M. Bauer M. Sommer C.. (2016). Reduced oxytocin receptor gene expression and binding sites in different brain regions in schizophrenia: a post-mortem study. Schizophr. Res. 177, 59–66. 10.1016/j.schres.2016.04.019 27132494

[B129] Unkelbach C. Guastella A. J. Forgas J. P. (2008). Oxytocin selectively facilitates recognition of positive sex and relationship words. Psychol. Sci. 19, 1092–1094. 10.1111/j.1467-9280.2008.02206.x 19076479

[B130] Unternaehrer E. Meyer A. H. Burkhardt S. C. Dempster E. Staehli S. Theill N.. (2015). Childhood maternal care is associated with DNA methylation of the genes for brain-derived neurotrophic factor (BDNF). and oxytocin receptor (OXTR). in peripheral blood cells in adult men and women. Stress 18, 451–461. 10.3109/10253890.2015.1038992 26061800

[B131] Valeeva G. Valiullina F. Khazipov R. (2013). Excitatory actions of GABA in the intact neonatal rodent hippocampus *in vitro*. Front. Cell. Neurosci. 7, 20. 10.3389/fncel.2013.00020 23467988 PMC3587803

[B132] Visscher P. M. Yengo L. Cox N. J. Wray N. R. (2021). Discovery and implications of polygenicity of common diseases. Science 373, 1468–1473. 10.1126/science.abi8206 34554790 PMC9945947

[B133] Volavka J. (2013). Violence in schizophrenia and bipolar disorder. Psychiatr. Danub. 25, 24–33. 23470603

[B134] Wang F. Yin X. S. Lu J. Cen C. Wang Y. (2022). Phosphorylation-dependent positive feedback on the oxytocin receptor through the kinase PKD1 contributes to long-term social memory. Sci. Signal 15, eabd0033. 10.1126/scisignal.abd0033 35104164

[B135] Wang J. Zhang P. Li W. Wen Q. Liu F. Xu J.. (2021). Right posterior insula and putamen volume mediate the effect of oxytocin receptor polygenic risk for autism spectrum disorders on reward dependence in healthy adults. Cereb. Cortex 31, 746–756. 10.1093/cercor/bhaa198 32710107

[B136] Watanabe T. Otowa T. Abe O. Kuwabara H. Aoki Y. Natsubori T.. (2017). Oxytocin receptor gene variations predict neural and behavioral response to oxytocin in autism. Soc. Cogn. Affect. Neurosci. 12, 496–506. 10.1093/scan/nsw150 27798253 PMC5390696

[B137] Wei J. Ma L. Ju P. Yang B. Wang Y. X. Chen J. (2020). Involvement of oxytocin receptor/Erk/MAPK SIgnaling in the mPFC in early life stress-induced autistic-like behaviors. Front. Cell Dev. Biol. 8, 564485. 10.3389/fcell.2020.564485 33134294 PMC7561716

[B138] Winslow J. T. Insel T. R. (2002). The social deficits of the oxytocin knockout mouse. Neuropeptides 36, 221–229. 10.1054/npep.2002.0909 12359512

[B139] Womersley J. S. Hemmings S. M. J. Ziegler C. Gutridge A. Ahmed-Leitao F. Rosenstein D.. (2020). Childhood emotional neglect and oxytocin receptor variants: association with limbic brain volumes. World J. Biol. Psychiatry 21, 513–528. 10.1080/15622975.2019.1584331 30806136

[B140] Wu S. Jia M. Ruan Y. Liu J. Guo Y. Shuang M.. (2005). Positive association of the oxytocin receptor gene (OXTR). with autism in the Chinese Han population. Biol. Psychiatry 58, 74–77. 10.1016/j.biopsych.2005.03.013 15992526

[B141] Yamasue H. Okada T. Munesue T. Kuroda M. Fujioka T. Uno Y.. (2020). Effect of intranasal oxytocin on the core social symptoms of autism spectrum disorder: a randomized clinical trial. Mol. Psychiatry 25, 1849–1858. 10.1038/s41380-018-0097-2 29955161

[B142] Yatawara C. J. Einfeld S. L. Hickie I. B. Davenport T. A. Guastella A. J. (2016). The effect of oxytocin nasal spray on social interaction deficits observed in young children with autism: a randomized clinical crossover trial. Mol. Psychiatry 21, 1225–1231. 10.1038/mp.2015.162 26503762 PMC4995545

[B143] Yoshida M. Takayanagi Y. Inoue K. Kimura T. Young L. J. Onaka T.. (2009). Evidence that oxytocin exerts anxiolytic effects *via* oxytocin receptor expressed in serotonergic neurons in mice. J. Neurosci. 29, 2259–2271. 10.1523/JNEUROSCI.5593-08.2009 19228979 PMC6666325

[B144] Zhang M. Liu N. Chen H. Zhang N. (2020). Oxytocin receptor gene, childhood maltreatment and borderline personality disorder features among male inmates in China. BMC Psychiatry 20, 332. 10.1186/s12888-020-02710-0 32580785 PMC7315490

[B145] Zhong M. Yang M. Sanborn B. M. (2003). Extracellular signal-regulated kinase 1/2 activation by myometrial oxytocin receptor involves Galpha(q)Gbetagamma and epidermal growth factor receptor tyrosine kinase activation. Endocrinology 144, 2947–2956. 10.1210/en.2002-221039 12810550

[B146] Zingg H. H. Laporte S. A. (2003). The oxytocin receptor. Trends Endocrinol. Metab. 14, 222–227. 10.1016/S1043-2760(03)00080-8 12826328

